# Supplementary material for: Forecasting individual progression trajectories in Alzheimer’s disease
Source: Nat Commun. 2023 Feb 10;14:761. doi: 10.1038/s41467-022-35712-5 (PMC9918533; doi:10.1038/s41467-022-35712-5)
Supplement: Supplementary file 1 — Supplementary Information [file 41467_2022_35712_MOESM1_ESM.pdf]

FORECASTING INDIVIDUAL PROGRESSION TRAJECTORIES IN ALZHEIMER'S DISEASE

Etienne Maheux, Igor Koval, Juliette Ortholand, Colin Birkenbihl, Damiano Archetti, Vincent Bouteloup,

Stéphane Epelbaum, Carole Dufouil, Martin Hofmann-Apitius, Stanley Durrleman

Supplementary information

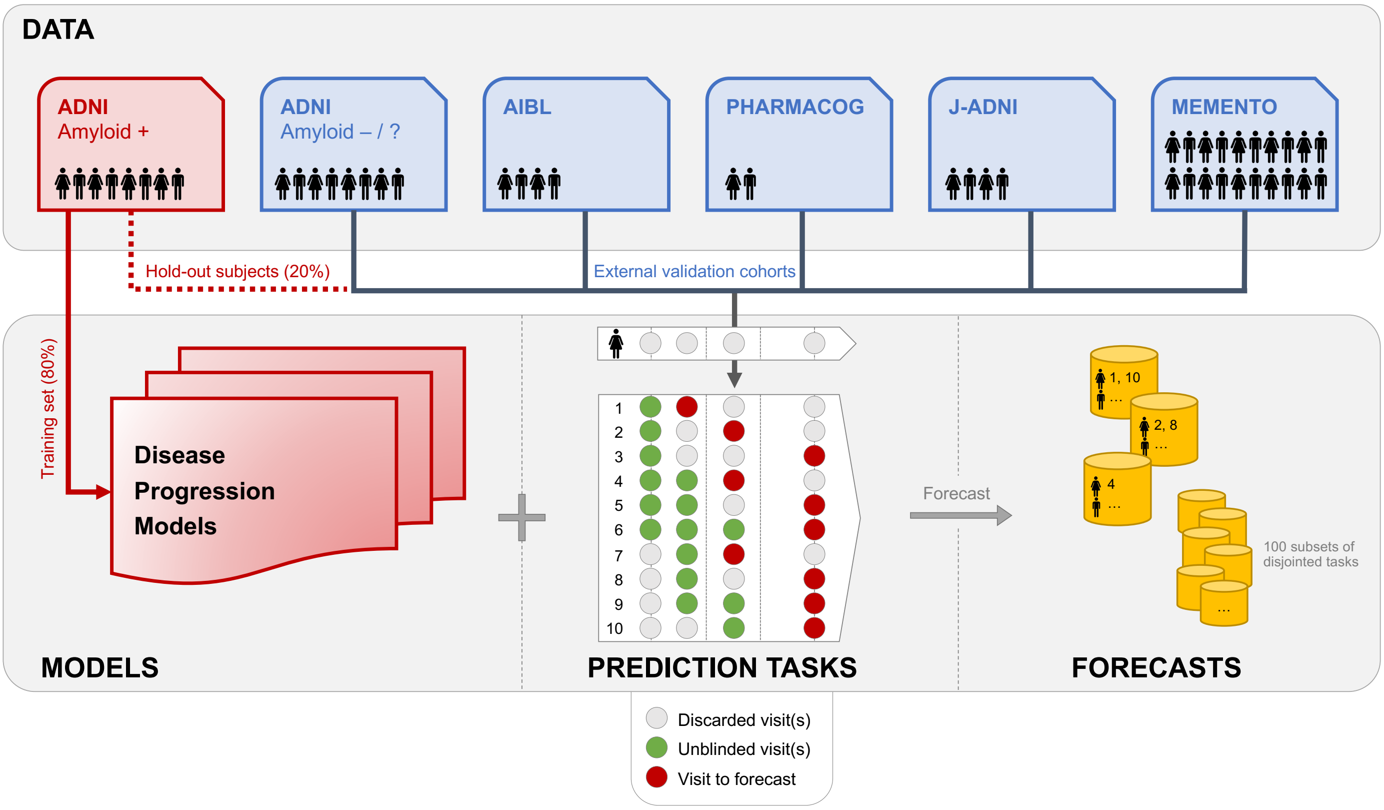

Supplementary Figure 1 Experimental protocol. Ten models were trained on 80% of the amyloid-positive participants from ADNI. Models were tested on hold-out amyloid positive ADNI participants, ADNI participants with unknown or negative amyloid status and participants from four external cohorts. For each test subject, a series of forecasts was made by partially blinding some of his visits. In this example, 10 forecasts are derived from a single participant having four visits. These predictions are split randomly into different sets, such that, in each set, the forecasts deriving from a given subject never share common visits. In this example, forecast 1 and 2 share a common visit: they are distributed in different sets, whereas forecasts 1 and 10 may be in the same set.

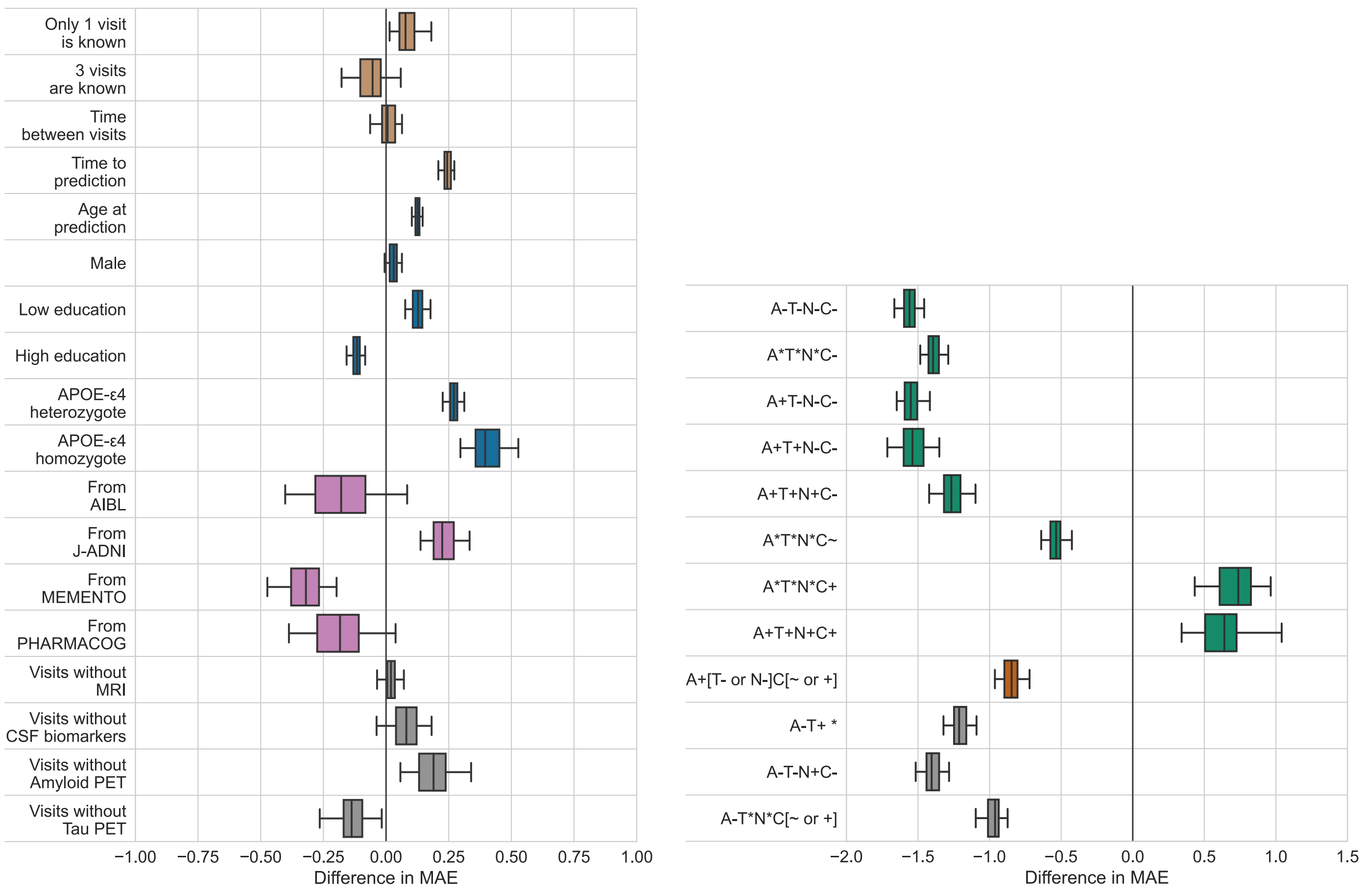

Supplementary Figure 2 Forecast of mini-mental state examination (MMSE) with AD Course Map: changes in absolute errors due to covariates. Left panel: changes due to forecast design (4 top rows, in brown), genetic and sociodemographic characteristics of the participant (rows 5 to 10, in blue), the cohort of the participant (rows 11 to 14, in pink), and missing data (rows 15 to 18, in gray). Right panel: changes due to A(myloid)/T(au)/N(eurodegeneration)/C(linical) status of the participant, grouped in: Alzheimer's continuum at the top (8 top rows, in green), possible Alzheimer's disease and concomitant non-Alzheimer's pathologic change in between (row 9, in orange), suspected non-Alzheimer's pathophysiology (SNAP) at the bottom (3 bottom row, in gray). A positive difference in mean absolute error (MAE) means forecasts are less accurate than for the reference participant (A+T+N+C~) and design. Box plots represent median value, first and third quartiles; whiskers represent the empirical 95% confidence interval. Statistics are computed for n=100 resampling of the validation set (see Methods). Source data are provided as a Source Data file. MAE: mean absolute error.

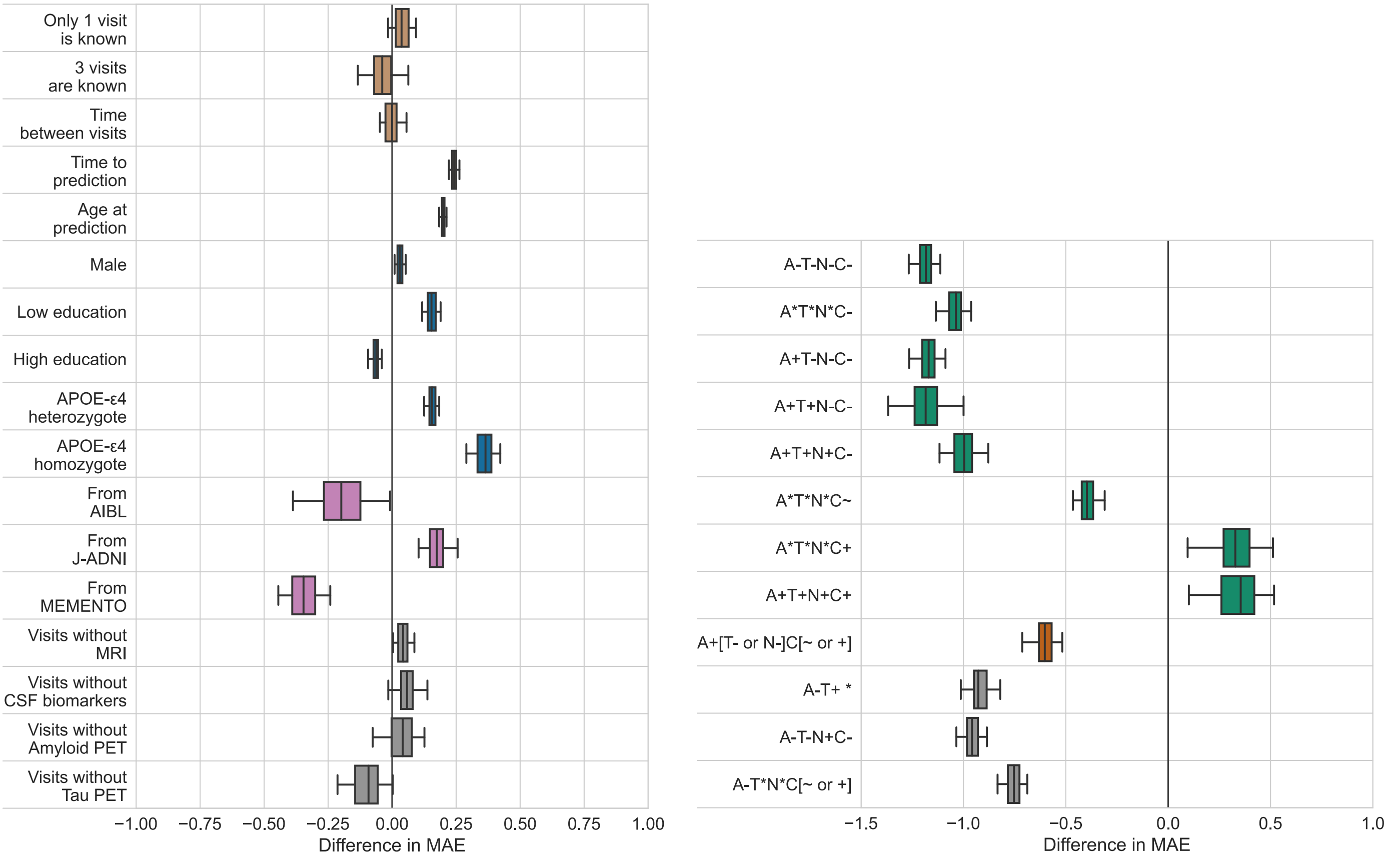

Supplementary Figure 3 Forecast of the clinical dementia rating – sum of boxes (CDR-SB) with AD Course Map: changes in absolute errors due to covariates. Left panel: changes due to forecast design (4 top rows, in brown), genetic and sociodemographic characteristics of the participant (rows 5 to 10, in blue), the cohort of the participant (rows 11 to 13, in pink), and missing data (rows 14 to 17, in gray). Right panel, changes due to A(myloid)/T(au)/N(eurodegeneration)/C(linical) status of the participant, grouped in: Alzheimer's continuum at the top (8 top rows, in green), possible Alzheimer's disease and concomitant non-Alzheimer's pathologic change in between (row 9, in orange), suspected non-Alzheimer's pathophysiology (SNAP) at the bottom (3 bottom row, in gray). A positive difference in mean absolute error (MAE) means forecasts are less accurate than for the reference participant (A+T+N+C~) and design. Box plots represent median value, first and third quartiles; whiskers represent the empirical 95% confidence interval. Statistics are computed for n=100 resampling of the validation set (see Methods). Source data are provided as a Source Data file. MAE: mean absolute error.

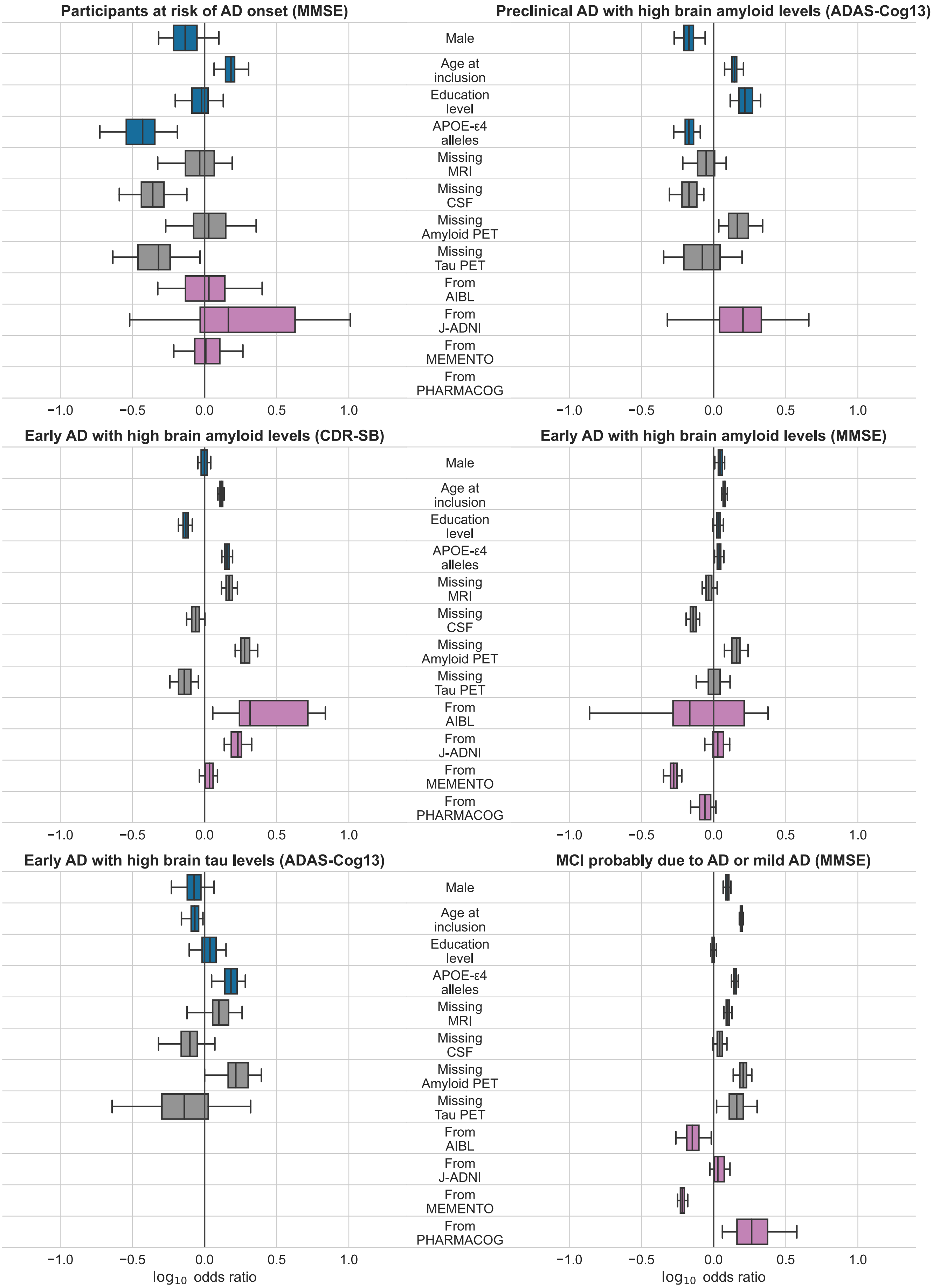

Supplementary Figure 4 Analysis of the biases in the population selected by AD Course Map as likely to be progressors for the considered outcome. For each panel, the first 4 rows (in blue) correspond to genetic and sociodemographic characteristics of the participant; rows 5 to 8 (in gray) correspond to missing data, and the last 4 rows (in pink) correspond to the cohort of the participant. Box plots represent median value, first and third quartiles; whiskers represent the empirical 95% confidence interval. Statistics are computed for n=100 resampling of the validation set (see Methods). Source data are provided as a Source Data file.

Feature	AD Course Map	RNN-AD
ADAS-Cog13	4.07 ± 0.20 [3.88, 4.35]	7.71 ± 0.72 [7.07, 8.28]
MMSE	1.71 ± 0.04 [1.67, 1.74]	3.15 ± 0.23 [2.91, 3.38]
CDR-SB	0.95 ± 0.11 [0.82, 1.07]	2.31 ± 0.23 [1.99, 2.57]
Lateral ventricles volume (% ICV)	0.16 ± 0.01 [0.14, 0.17]	0.66 ± 0.13 [0.56, 0.75]
Hippocampus volume (‰ ICV)	0.12 ± 0.00 [0.12, 0.12]	0.33 ± 0.04 [0.28, 0.37]
Abeta$_{1-42}$ level in CSF (§)	0.47 ± 0.02 [0.45, 0.49]	0.37 ± 0.08 [0.29, 0.48]
p-Tau$_{181}$ level in CSF (§)	0.69 ± 0.04 [0.65, 0.74]	0.51 ± 0.08 [0.43, 0.62]
PET Amyloid (CL)	8.13 ± 3.50 [5.46, 13.47]	22.67 ± 1.93 [20.54, 24.40]
PET Tau (SUVR)	0.24 ± 0.02 [0.22, 0.26]	0.29 ± 0.07 [0.21, 0.35]

Supplementary Table 1 Goodness-of-fit of AD Course Map and RNN-AD. Root mean square errors between the predicted and observed endpoint in the training data set. Data is presented as mean ± standard deviation, with empirical 80% confidence interval on the n=10 models trained. Source data are provided as a Source Data file. ICV: Intracranial volume. (§) In harmonized units (see Methods). CL: Centiloid Scale. SUVR: Standard Uptake Value Ratio.

Endpoint	Models compared (left – right)		Mean difference in adjusted MAE	Mean difference in adjusted MAE (relative)	P-value (empirical for n=100 runs)
ADAS-Cog13	AD Course Map	RNN-AD	-0.55	-8.5%	0.07
		Linear mixed model	-1.87	-23.9%	< 0.01
		No-change prediction	-2.87	-32.5%	< 0.01
	RNN-AD	Linear mixed model	-1.32	-16.8%	< 0.01
		No-change prediction	-2.32	-26.2%	< 0.01
	Linear mixed model	No-change prediction	-1.00	-11.3%	< 0.01
MMSE	AD Course Map	RNN-AD	-0.20	-7.4%	0.01
		Linear mixed model	-0.78	-23.4%	< 0.01
		No-change prediction	-0.99	-28.1%	< 0.01
	RNN-AD	Linear mixed model	-0.57	-17.2%	< 0.01
		No-change prediction	-0.79	-22.3%	< 0.01
	Linear mixed model	No-change prediction	-0.22	-6.2%	< 0.01
CDR-SB	AD Course Map	RNN-AD	-0.10	-4.9%	0.12
		Linear mixed model	-0.50	-21.1%	< 0.01
		No-change prediction	-0.67	-26.6%	< 0.01
	RNN-AD	Linear mixed model	-0.40	-17.0%	< 0.01
		No-change prediction	-0.58	-22.8%	< 0.01
	Linear mixed model	No-change prediction	-0.18	-7.0%	< 0.01

Supplementary Table 2 Comparison of forecast errors across models. The adjusted mean absolute error (MAE) is compared for all model pairs. AD Course Map significantly outperforms all alternative forecasting methods but RNN-AD for CDR-SB and ADAS-Cog13. Statistics are computed for n=100 resampling of the validation set (see Methods). P-value is calculated from the empirical distribution of adjusted MAE paired differences (non-parametric two-sided test, p-values under 0.01 could not be estimated); no multiple comparison adjustment is performed. Source data are provided as a Source Data file (same data as for the Figure 2).

Trial	RNN-AD	AD Course Map
Participants at risk of AD onset (MMSE)	29.3 ± 8.3 [11.5, 41.6]	37.8 ± 7.0 [21.2, 48.1]
Preclinical AD with high brain amyloid levels (ADAS-Cog13)	12.8 ± 3.1 [7.0, 18.7]	27.7 ± 3.5 [20.8, 34.7]
Early AD with high brain amyloid levels (CDR-SB)	34.0 ± 1.3 [31.9, 36.8]	35.0 ± 1.2 [33.1, 36.9]
Early AD with high brain amyloid levels (MMSE)	36.2 ± 1.3 [34.2, 39.1]	38.9 ± 1.4 [36.5, 41.9]
Early AD with high brain tau levels (ADAS-Cog13)	34.9 ± 3.2 [29.5, 41.4]	47.2 ± 2.7 [42.1, 51.7]
MCI probably due to AD or mild AD (MMSE)	32.0 ± 1.0 [30.4, 34.1]	44.4 ± 0.8 [42.7, 46.0]

Supplementary Table 3 Pearson correlations (in %) between true outcome and prognosis score, for each simulated trial. Prognosis scores derived from AD Course Map always outperform the ones derived from RNN-AD. Data are presented as mean value ± standard deviation, with empirical 95% confidence interval. Statistics are computed for n=100 resampling of the validation set (see Methods). Source data are provided as a Source Data file.

Trial	Target	Prevalence (%)	Selection	Selected (%)	Sensitivity (%)	Specificity (%)	Balanced Accuracy (%)	Precision (%)	NPV (%)	LR+	LR-	DOR
Participants at risk of AD onset (MMSE)	Patients with MMSE change greater than 0 pt in 4 years	67.5 ± 2.3 [63.0, 71.9]	AD Course Map	50.3 ± 2.3 [45.8, 55.0]	65.3 ± 3.2 [59.2, 71.8]	80.9 ± 3.6 [74.6, 87.5]	73.1 ± 2.7 [68.1, 77.7]	87.7 ± 2.4 [83.7, 92.0]	52.9 ± 4.1 [44.7, 61.5]	3.57 ± 0.80 [2.41, 5.17]	0.43 ± 0.05 [0.34, 0.52]	8.55 ± 2.66 [4.66, 14.73]
			RNN-AD	50.6 ± 2.7 [45.2, 56.3]	66.0 ± 3.1 [60.5, 71.9]	81.4 ± 4.2 [73.8, 89.5]	73.7 ± 2.7 [68.7, 79.4]	88.1 ± 2.8 [82.8, 93.6]	53.6 ± 3.7 [46.8, 59.4]	3.78 ± 1.02 [2.43, 6.43]	0.42 ± 0.05 [0.33, 0.51]	9.32 ± 3.37 [5.00, 18.39]
Preclinical AD with high brain amyloid levels (ADAS-Cog13)	Patients with ADAS-Cog13 change greater than 1.9 pts in 4 years	49.8 ± 1.5 [47.3, 52.9]	APOE-ε4 carriers	41.4 ± 0.8 [40.3, 43.0]	45.6 ± 1.7 [42.0, 49.0]	62.7 ± 1.9 [59.3, 66.1]	54.2 ± 1.6 [51.4, 57.4]	54.8 ± 2.3 [50.3, 59.0]	53.8 ± 2.2 [49.6, 57.6]	1.23 ± 0.10 [1.07, 1.43]	0.87 ± 0.05 [0.78, 0.95]	1.43 ± 0.19 [1.12, 1.84]
			AD Course Map	49.4 ± 1.5 [45.7, 52.3]	58.6 ± 2.3 [54.4, 62.4]	59.7 ± 2.2 [55.5, 64.0]	59.1 ± 1.6 [55.8, 62.1]	59.1 ± 2.1 [54.8, 63.5]	59.2 ± 2.2 [55.3, 63.1]	1.46 ± 0.10 [1.26, 1.64]	0.69 ± 0.05 [0.61, 0.79]	2.12 ± 0.28 [1.59, 2.68]
			RNN-AD	49.9 ± 1.5 [47.2, 53.4]	53.0 ± 2.2 [49.1, 57.2]	53.3 ± 2.6 [48.1, 57.7]	53.2 ± 1.9 [49.2, 56.4]	53.0 ± 2.8 [47.6, 58.9]	53.3 ± 2.0 [49.5, 57.3]	1.14 ± 0.09 [0.97, 1.31]	0.88 ± 0.07 [0.78, 1.03]	1.30 ± 0.20 [0.94, 1.67]
Early AD with high brain amyloid levels (CDR-SB)	Patients with CDR-SB change greater than 0.5 pt in 1.5 years	51.5 ± 0.8 [50.3, 53.0]	APOE-ε4 carriers	62.2 ± 0.4 [61.5, 63.0]	66.7 ± 0.7 [65.5, 68.2]	42.7 ± 0.9 [40.9, 44.1]	54.7 ± 0.7 [53.3, 55.9]	55.3 ± 1.0 [53.7, 57.3]	54.7 ± 1.1 [52.7, 57.0]	1.16 ± 0.03 [1.11, 1.21]	0.78 ± 0.03 [0.73, 0.84]	1.50 ± 0.09 [1.33, 1.65]
			AD Course Map	52.3 ± 0.6 [51.4, 53.5]	66.2 ± 1.0 [64.7, 68.0]	62.4 ± 1.0 [60.5, 64.4]	64.3 ± 0.8 [62.8, 65.9]	65.1 ± 1.0 [63.0, 67.1]	63.5 ± 1.2 [61.3, 65.8]	1.76 ± 0.06 [1.66, 1.87]	0.54 ± 0.02 [0.51, 0.58]	3.26 ± 0.22 [2.85, 3.73]
			RNN-AD	50.7 ± 0.6 [49.5, 51.9]	64.8 ± 1.0 [62.7, 66.6]	64.2 ± 1.2 [61.7, 66.4]	64.5 ± 0.9 [62.8, 66.1]	65.8 ± 1.2 [63.4, 67.8]	63.2 ± 1.2 [60.9, 65.4]	1.81 ± 0.07 [1.68, 1.94]	0.55 ± 0.02 [0.51, 0.59]	3.32 ± 0.26 [2.86, 3.79]
Early AD with high brain amyloid levels (MMSE)	Patients with MMSE change greater than 1.1 pts in 1.5 years	49.6 ± 0.7 [48.2, 50.8]	APOE-ε4 carriers	62.4 ± 0.4 [61.8, 63.2]	64.7 ± 0.9 [62.9, 66.6]	39.9 ± 0.9 [38.2, 41.4]	52.3 ± 0.8 [50.7, 53.9]	51.4 ± 0.9 [49.9, 53.3]	53.5 ± 1.3 [50.9, 55.9]	1.08 ± 0.03 [1.02, 1.13]	0.89 ± 0.04 [0.81, 0.96]	1.22 ± 0.08 [1.06, 1.40]
			AD Course Map	49.6 ± 0.6 [48.7, 50.6]	63.6 ± 0.9 [62.0, 65.2]	64.1 ± 1.0 [62.4, 66.0]	63.8 ± 0.7 [62.4, 65.0]	63.5 ± 1.0 [61.4, 65.3]	64.2 ± 1.0 [62.5, 66.2]	1.77 ± 0.06 [1.67, 1.88]	0.57 ± 0.02 [0.54, 0.60]	3.12 ± 0.20 [2.76, 3.46]
			RNN-AD	50.6 ± 0.7 [49.2, 52.0]	63.2 ± 1.0 [61.1, 65.1]	61.7 ± 1.1 [59.3, 63.4]	62.4 ± 0.8 [60.7, 63.8]	61.8 ± 1.1 [59.6, 63.8]	63.1 ± 1.1 [61.2, 65.5]	1.65 ± 0.06 [1.52, 1.75]	0.60 ± 0.02 [0.56, 0.64]	2.77 ± 0.19 [2.39, 3.10]
Early AD with high brain tau levels (ADAS-Cog13)	Patients with ADAS-Cog13 change greater than 6.1 pts in 4.5 years	50.2 ± 2.0 [46.5, 53.7]	APOE-ε4 carriers	57.7 ± 1.9 [54.5, 61.7]	70.1 ± 2.6 [65.4, 74.6]	54.7 ± 2.3 [50.4, 58.8]	62.4 ± 1.7 [59.0, 65.5]	60.9 ± 2.6 [55.5, 65.5]	64.5 ± 2.5 [60.3, 69.5]	1.55 ± 0.10 [1.37, 1.74]	0.55 ± 0.05 [0.46, 0.64]	2.88 ± 0.43 [2.12, 3.70]
			AD Course Map	49.3 ± 2.0 [45.1, 53.3]	69.5 ± 2.8 [63.4, 74.5]	71.0 ± 2.8 [65.7, 76.5]	70.3 ± 2.1 [66.4, 74.2]	70.7 ± 2.8 [66.1, 76.6]	69.8 ± 2.7 [64.8, 75.1]	2.42 ± 0.27 [1.99, 3.09]	0.43 ± 0.04 [0.35, 0.52]	5.74 ± 1.18 [3.90, 8.40]
			RNN-AD	49.5 ± 2.0 [46.3, 53.9]	68.8 ± 2.6 [64.3, 73.7]	69.9 ± 2.6 [65.2, 74.8]	69.4 ± 1.8 [66.2, 72.6]	69.7 ± 2.8 [65.0, 75.0]	69.0 ± 2.3 [64.8, 73.5]	2.31 ± 0.23 [1.94, 2.78]	0.45 ± 0.04 [0.38, 0.52]	5.24 ± 0.92 [3.83, 7.02]
MCI probably due to AD or mild AD (MMSE)	Patients with MMSE change greater than 0.9 pt in 3 years	50.4 ± 0.3 [49.7, 51.0]	APOE-ε4 carriers	39.1 ± 0.2 [38.7, 39.4]	48.1 ± 0.4 [47.3, 48.8]	70.1 ± 0.4 [69.5, 71.1]	59.1 ± 0.4 [58.5, 59.9]	62.1 ± 0.6 [61.0, 63.3]	57.1 ± 0.5 [56.1, 57.9]	1.61 ± 0.04 [1.56, 1.68]	0.74 ± 0.01 [0.72, 0.76]	2.18 ± 0.08 [2.06, 2.33]
			AD Course Map	49.2 ± 0.3 [48.6, 49.9]	63.6 ± 0.6 [62.4, 64.5]	65.4 ± 0.5 [64.3, 66.5]	64.5 ± 0.4 [63.6, 65.3]	65.2 ± 0.5 [64.2, 66.1]	63.9 ± 0.6 [62.7, 65.0]	1.84 ± 0.04 [1.77, 1.91]	0.56 ± 0.01 [0.54, 0.58]	3.31 ± 0.13 [3.06, 3.54]
			RNN-AD	44.7 ± 0.4 [44.1, 45.4]	54.6 ± 0.6 [53.5, 55.6]	65.4 ± 0.6 [64.3, 66.5]	60.0 ± 0.5 [59.0, 60.8]	61.6 ± 0.6 [60.6, 62.6]	58.6 ± 0.5 [57.4, 59.6]	1.58 ± 0.04 [1.51, 1.64]	0.69 ± 0.01 [0.67, 0.72]	2.28 ± 0.09 [2.09, 2.44]

Supplementary Table 4 Classification metrics for the identification of fast progressors at entry into a trial. Data are presented as mean value ± SD, with empirical 95% CI. Statistics are computed for n=100 resampling of the validation set (see Methods). Source data are provided as a Source Data file. NPV: negative predictive value, LR: likelihood ratio, DOR: diagnostic odds ratio.

Trial	APOE-ε4 carriers	Selection with RNN-AD	Selection with AD Course Map
Participants at risk of AD onset (MMSE)	/	-40.6 ± 7.2 [-53.6, -28.0]	-50.2 ± 7.1 [-62.3, -33.3]
Preclinical AD with high brain amyloid levels (ADAS-Cog13)	-6.4 ± 5.8 [-16.2, 4.9]	-20.5 ± 8.2 [-34.0, -5.6]	-40.9 ± 4.9 [-49.0, -30.1]
Early AD with high brain amyloid levels (CDR-SB)	-13.0 ± 1.8 [-16.3, -9.0]	-38.1 ± 1.7 [-41.4, -34.3]	-38.1 ± 1.6 [-40.9, -35.1]
Early AD with high brain amyloid levels (MMSE)	-5.2 ± 2.3 [-9.4, -0.2]	-42.1 ± 2.2 [-45.8, -37.6]	-45.4 ± 2.0 [-49.3, -41.3]
Early AD with high brain tau levels (ADAS-Cog13)	-24.5 ± 3.4 [-30.5, -18.0]	-41.2 ± 3.9 [-49.5, -34.7]	-44.6 ± 3.9 [-52.2, -37.5]
MCI probably due to AD or mild AD (MMSE)	-37.7 ± 0.9 [-39.5, -35.9]	-37.3 ± 1.1 [-39.2, -34.6]	-43.1 ± 0.8 [-44.7, -41.4]

Supplementary Table 5 Sample size ratio (in %) of enriched trials compared to sample size of trial without enrichment for a theoretical treatment effect of 25%. Enrichment with AD Course Map always outperforms enrichment based on alternatives; the corresponding sample size reductions range from 38% to 50%. Data are presented as mean value ± standard deviation, with empirical 95% confidence interval. Statistics are computed for n=100 resampling of the validation set (see Methods). Source data are provided as a Source Data file (the same data as for the Figure 6).

Biomarker	ADNI	AIBL	J-ADNI	MEMENTO	PHARMACOG
Cortical-summary SUVR on Amyloid PET (Florbetapir)	$300.66\,x - 208.84$	/	/	/	/
Cortical-summary SUVR on Amyloid PET (Florbetaben)	$244.20\,x - 170.80$	/	/	/	/
Abeta$_{1\text{-}42}$ level in CSF	$\frac{x - 1256.69}{536.74}$	$\frac{x - 592.77}{238.71}$	$\frac{x - 396.82}{125.04}$	$\frac{x - 1082.91}{365.57}$	$\frac{x - 816.72}{249.11}$
p-Tau$_{181}$ level in CSF	$\frac{x - 24.17}{13.56}$	$\frac{x - 69.56}{23.86}$	$\frac{x - 52.25}{21.08}$	$\frac{x - 63.41}{29.41}$	$\frac{x - 63.93}{34.19}$
Total Tau level in CSF	$\frac{x - 257.53}{122.93}$	$\frac{x - 530.07}{236.23}$	/	$\frac{x - 399.06}{268.13}$	$\frac{x - 439.96}{338.91}$

Supplementary Table 6 Harmonization equations for CSF and Amyloid PET biomarkers when available. The CSF equations result from the cohort-wise adjustment model described in Methods section.

Biomarker group	Biomarker	Positivity cutoff
Amyloid	Abeta$_{1\text{-}42}$ level in CSF	< -0.5 (§)
	Cortical summary SUVR on Amyloid PET	> 30 CL
Tau	p-Tau$_{181}$ level in CSF	> -0.1 (§)
	Cortical summary SUVR on Tau PET	> 1.55
Neurodegeneration	Total Tau level in CSF	> 0 (§)
	Normalized hippocampus volume	< 0.47 (% ICV)
Cognition / Clinical	CDR Global	C– [CDR = 0]<br>C~ [CDR = 0.5]<br>C+ [CDR ≥ 1]

Supplementary Table 7 Biomarker cutoff points for defining A(myloid)/T(au)/N(eurodegeneration)/C(linical) profiles. For biomarker groups with multiple possible biomarkers, we considered the group to be positive if at least one biomarker was positive. We grouped very rare A/T/N/C combinations (less than 0.5%) together or under more frequent profiles. (§) Value in harmonized units (see Methods). CL: Centiloid Scale. ICV: Intracranial volume.

Supplementary Table 8 Characteristics of participants included in the six simulated trials. ARC: annual rate of change. N.C.: not disclosed. Format for non-constant continuous variables: mean ± standard deviation [95% confidence interval] (% available).

(a) Participants at risk of AD onset (MMSE)

	Pooled	ADNI	AIBL	J-ADNI	MEMENTO
Number of forecasts	197	110	19	5	63
Number of subjects	69	38	9	5	17
Number of forecasts per subject	2.9 ± 1.9 [1, 6]	2.9 ± 2.0 [1, 6]	2.1 ± 1.1 [1, 3]	1.0 ± 0.0 [1, 1]	3.7 ± 1.8 [1, 6]
Trial duration (y)	3.6 ± 0.6 [3.0, 4.8]	3.8 ± 0.6 [3.0, 4.9]	3.4 ± 0.7 [3.0, 4.5]	3.0 ± 0.0 [3.0, 3.0]	3.5 ± 0.4 [3.0, 4.2]
Age at screening	70.1 ± 4.2 [61.7, 75.9]	71.0 ± 3.3 [65.4, 75.9]	65.9 ± 3.0 [62.0, 71.5]	65.2 ± 4.3 [60.1, 68.9]	70.3 ± 5.0 [61.1, 75.9]
Female	64.0%	60.9%	57.9%	40.0%	73.0%
**Education level**					
≤ 9 years	7.6%	0.9%	15.8%		17.5%
Between 10 and 15 years	41.6%	35.5%	63.2%	60.0%	44.4%
≥ 16 years	49.7%	63.6%	21.1%	40.0%	34.9%
Missing	1.0%				3.2%
**APOE-ε4 copies**					
1	73.1%	91.8%	21.1%	60.0%	57.1%
2	26.9%	8.2%	78.9%	40.0%	42.9%
**A/T/N/C profile at inclusion**					
A-T-N-C-	4.1%	1.8%			9.5%
A*T*N*C-	18.3%	1.8%	78.9%	40.0%	27.0%
A+T-N-C-	49.2%	55.5%	15.8%	60.0%	47.6%
A+T+N-C-	12.7%	20.0%	5.3%		3.2%
A+T+N+C-	15.7%	20.9%			12.7%
**Score distribution at inclusion**					
CDR (global)			0		
CDR-SB	0.0 ± 0.1 [0.0, 0.5] (97.5 %)	0.0 ± 0.1 [0.0, 0.5]	0.0 ± 0.0 [0.0, 0.0]	N.C.	0.1 ± 0.2 [0.0, 0.5]
MMSE	28.9 ± 1.1 [26.0, 30.0]	28.9 ± 1.1 [27.0, 30.0]	28.8 ± 1.2 [26.4, 30.0]	28.8 ± 1.6 [26.3, 30.0]	28.8 ± 1.1 [26.6, 30.0]
ADAS-Cog13	8.2 ± 4.1 [1.6, 15.7] (55.8 %)	8.2 ± 4.1 [1.6, 15.7]	/	N.C.	/
**ARC distribution**					
Annual rate of change CDR-SB	0.1 ± 0.3 [0.0, 0.9] (97.0 %)	0.2 ± 0.3 [0.0, 1.1] (94.5 %)	0.0 ± 0.0 [0.0, 0.0]	0.1 ± 0.2 [0.0, 0.4]	0.1 ± 0.2 [0.0, 0.6]
Annual rate of change MMSE	-0.1 ± 0.5 [-1.7, 0.7]	-0.2 ± 0.6 [-1.7, 0.7]	0.1 ± 0.5 [-1.0, 0.7]	-0.5 ± 0.8 [-1.6, 0.3]	-0.1 ± 0.5 [-1.5, 0.6]
Annual rate of change ADAS-Cog13	0.6 ± 1.6 [-1.3, 4.3] (56.9 %)	0.6 ± 1.5 [-1.3, 4.0] (97.3 %)	/	0.6 ± 2.4 [-1.2, 4.3]	/

(b) Preclinical AD with high brain amyloid levels (ADAS-Cog13)

	Pooled	ADNI	J-ADNI
Number of forecasts	434	426	8
Number of subjects	126	118	8
Number of forecasts per subject	3.4 ± 2.9 [1, 12]	3.6 ± 2.9 [1, 12]	1.0
Trial duration (y)	3.9 ± 0.6 [3.0, 5.0]	3.9 ± 0.6 [3.0, 5.0]	3.0 ± 0.0 [3.0, 3.0]
Age at screening	74.8 ± 4.4 [65.4, 80.8]	74.9 ± 4.3 [65.7, 80.8]	67.9 ± 5.2 [61.5, 77.2]
Female	53.9 %	54.2 %	37.5 %
**Education level**			
≤ 9 years	1.6%	1.6%	
Between 10 and 15 years	30.2%	29.8%	50.0%
≥ 16 years	68.2%	68.5%	50.0%
**APOE-ε4 copies**			
0	59.4%	59.6%	50.0%
1	36.9%	36.6%	50.0%
2	3.7%	3.8%	
**A/T/N/C profile at inclusion**			
A+T-N-C-	53.7%	53.3%	75.0%
A+T+N-C-	15.0%	15.0%	12.5%
A+T+N+C-	31.3%	31.7%	12.5%
**Score distribution at inclusion**			
CDR (global)		0	
CDR-SB	0.1 ± 0.2 [0.0, 0.5] (98.2 %)	0.1 ± 0.2 [0.0, 0.5]	N.C.
MMSE	29.2 ± 0.9 [27.0, 30.0]	29.2 ± 0.9 [27.0, 30.0]	29.2 ± 0.7 [28.2, 30.0]
ADAS-Cog13	8.3 ± 4.2 [1.0, 16.8] (98.2 %)	8.3 ± 4.2 [1.0, 16.8]	N.C.
**ARC distribution**			
Annual rate of change CDR-SB	0.2 ± 0.3 [0.0, 1.1] (96.5 %)	0.2 ± 0.3 [0.0, 1.1] (96.5 %)	0.0 ± 0.0 [0.0, 0.0]
Annual rate of change MMSE	-0.2 ± 0.6 [-1.3, 0.6] (99.5 %)	-0.2 ± 0.6 [-1.4, 0.6] (99.5 %)	-0.0 ± 0.5 [-0.9, 0.6]
Annual rate of change ADAS-Cog13	0.7 ± 1.5 [-1.5, 3.8]	0.7 ± 1.5 [-1.5, 3.9]	-0.4 ± 1.0 [-1.4, 1.5]

(c) Early AD with high brain amyloid levels (MMSE)

	Pooled	ADNI	AIBL	J-ADNI	MEMENTO	PHARMACOG
Number of forecasts	3,371	1,872	6	371	709	413
Number of subjects	895	581	5	66	167	76
Number of forecasts per subject	3.8 ± 2.6<br>[1, 10]	3.2 ± 2.2<br>[1, 8]	1.2 ± 0.4<br>[1, 2]	5.6 ± 2.8<br>[1, 9]	4.2 ± 3.0<br>[1, 11]	5.4 ± 2.8<br>[1, 12]
Trial duration (y)	1.4 ± 0.4<br>[1.0, 2.0]	1.4 ± 0.4<br>[1.0, 2.0]	1.5 ± 0.0<br>[1.5, 1.5]	1.4 ± 0.4<br>[1.0, 2.0]	1.4 ± 0.3<br>[1.0, 2.0]	1.3 ± 0.4<br>[1.0, 2.0]
Age at screening	73.3 ± 6.7<br>[59.5, 84.5]	74.0 ± 6.7<br>[59.7, 84.8]	76.9 ± 3.8<br>[72.2, 82.6]	72.8 ± 5.6<br>[62.0, 83.0]	73.0 ± 7.0<br>[56.3, 83.7]	71.0 ± 6.3<br>[57.2, 82.5]
Female	45.6%	41.0%	50.0%	46.1%	50.2%	57.9%
**Education level**						
≤ 9 years	11.7%	0.8%	50.0%	11.1%	24.0%	40.2%
Between 10 and 15 years	38.5%	33.1%	16.7%	52.3%	49.6%	32.0%
≥ 16 years	49.3%	66.1%	33.3%	36.7%	24.3%	27.8%
Missing	0.4%				2.1%	
**APOE-ε4 copies**						
0	36.0%	39.2%	50.0%	32.9%	33.0%	29.1%
1	48.7%	44.7%	33.3%	54.2%	52.3%	55.9%
2	13.6%	15.8%	16.7%	12.9%	9.3%	12.1%
Missing	1.7%	0.3%			5.4%	2.9%
**A/T/N/C profile at inclusion**						
A+T-N-C~	17.9%	24.1%		7.0%	9.4%	14.3%
A+T-N+C~	12.5%	8.2%		15.1%	16.8%	22.5%
A+T+N-C~	10.5%	13.8%	33.3%	8.1%	3.1%	10.2%
A+T+N+C~	56.8%	52.8%	33.3%	69.0%	63.5%	53.0%
A+T*N*C~	2.3%	1.1%	33.3%	0.8%	7.2%	
**Score distribution at inclusion**						
CDR (global)	0.5					
CDR-SB	1.5 ± 1.0<br>[0.0, 4.0]<br>(76.7 %)	1.6 ± 1.1<br>[0.0, 4.0]	2.2 ± 1.8<br>[0.1, 4.4]	N.C.	1.1 ± 0.9<br>[0.0, 3.5]	/
MMSE	27.3 ± 1.9<br>[24.0, 30.0]	27.5 ± 1.9<br>[24.0, 30.0]	26.5 ± 1.5<br>[25.0, 28.8]	26.4 ± 1.7<br>[24.0, 30.0]	27.4 ± 1.8<br>[24.0, 30.0]	26.6 ± 1.7<br>[24.0, 30.0]
ADAS-Cog13	17.2 ± 7.4<br>[4.0, 31.3]<br>(67.7 %)	16.6 ± 7.4<br>[3.7, 31.0]<br>(99.9 %)	/	N.C.	/	19.7 ± 6.6<br>[6.7, 33.9]<br>(99.8 %)
**ARC distribution**						
Annual rate of change CDR-SB	0.6 ± 1.1<br>[-1.0, 3.3]<br>(85.8 %)	0.5 ± 1.1<br>[-1.0, 3.1]<br>(98.9 %)	0.5 ± 1.1<br>[-0.9, 2.2]	0.9 ± 1.2<br>[-1.0, 3.8]	0.5 ± 1.1<br>[-0.9, 3.4]<br>(93.7 %)	/
Annual rate of change MMSE	-0.9 ± 2.0<br>[-5.6, 2.0]	-0.8 ± 2.0<br>[-5.5, 2.1]	-0.8 ± 1.8<br>[-3.2, 0.7]	-1.7 ± 1.9<br>[-5.9, 2.0]	-0.8 ± 1.9<br>[-5.3, 1.9]	-0.7 ± 2.0<br>[-6.0, 2.0]
Annual rate of change ADAS-Cog13	1.8 ± 4.3<br>[-5.5, 11.7]<br>(77.9 %)	1.7 ± 4.4<br>[-5.8, 12.0]<br>(99.0 %)	/	2.4 ± 3.7<br>[-4.3, 10.0]	/	1.6 ± 3.9<br>[-5.1, 11.0]<br>(97.1 %)

(d) Early AD with high brain amyloid levels (CDR-SB)

	Pooled	ADNI	AIBL	J-ADNI	MEMENTO
Number of forecasts	2,934	1,894	6	371	663
Number of subjects	824	589	5	66	164
Number of forecasts per subject	3.6 ± 2.5 [1, 9]	3.2 ± 2.3 [1, 9]	1.2 ± 0.4 [1, 2]	5.6 ± 2.8 [1, 9]	4.0 ± 2.9 [1, 10]
Trial duration (y)	1.4 ± 0.4 [1.0, 2.0]	1.4 ± 0.4 [1.0, 2.0]	1.5 ± 0.0 [1.5, 1.5]	1.4 ± 0.4 [1.0, 2.0]	1.4 ± 0.3 [1.0, 2.0]
Age at screening	73.7 ± 6.7 [59.6, 84.6]	74.1 ± 6.7 [59.9, 84.9]	76.9 ± 3.8 [72.2, 82.6]	72.8 ± 5.6 [62.0, 83.0]	72.9 ± 7.1 [56.2, 84.0]
Female	43.0%	40.2%	50.0%	46.1%	49.3%
**Education level**					
≤ 9 years	7.7%	0.8%	50.0%	11.1%	25.0%
Between 10 and 15 years	38.6%	32.9%	16.7%	52.3%	47.2%
≥ 16 years	53.2%	66.3%	33.3%	36.7%	25.5%
Missing	0.5%				2.3%
**APOE-ε4 copies**					
0	37.0%	38.9%	50.0%	32.9%	33.6%
1	47.4%	44.8%	33.3%	54.2%	51.3%
2	14.1%	15.9%	16.7%	12.9%	9.4%
Missing	1.5%	0.4%			5.7%
**A/T/N/C profile at inclusion**					
A+T-N-C~	18.5%	24.0%		7.0%	9.4%
A+T-N+C~	11.0%	7.9%		15.1%	17.5%
A+T+N-C~	10.8%	13.9%	33.3%	8.1%	3.3%
A+T+N+C~	57.1%	53.0%	33.3%	69.0%	62.3%
A+T*N*C~	2.7%	1.3%	33.3%	0.8%	7.5%
**Score distribution at inclusion**					
CDR (global)			0.5		
CDR-SB	1.5 ± 1.0 [0.0, 4.0] (87.4 %)	1.6 ± 1.1 [0.0, 4.0]	2.2 ± 1.8 [0.1, 4.4]	N.C.	1.1 ± 0.9 [0.0, 3.5]
MMSE	27.3 ± 1.9 [24.0, 30.0]	27.5 ± 1.9 [24.0, 30.0]	26.5 ± 1.5 [25.0, 28.8]	26.4 ± 1.7 [24.0, 30.0]	27.4 ± 1.8 [24.0, 30.0]
ADAS-Cog13	16.7 ± 7.5 [3.7, 31.2] (64.5 %)	16.7 ± 7.5 [3.7, 31.2] (99.9 %)	/	N.C.	/
**ARC distribution**					
Annual rate of change CDR-SB	0.6 ± 1.1 [-1.0, 3.3]	0.6 ± 1.1 [-1.0, 3.3]	0.5 ± 1.1 [-0.9, 2.2]	0.9 ± 1.2 [-1.0, 3.8]	0.5 ± 1.1 [-1.0, 3.6]
Annual rate of change MMSE	-1.0 ± 2.0 [-5.5, 2.0] (98.1 %)	-0.8 ± 2.0 [-5.5, 2.1] (97.4 %)	-0.8 ± 1.8 [-3.2, 0.7]	-1.7 ± 1.9 [-5.9, 2.0]	-0.9 ± 1.9 [-5.2, 1.9] (98.8 %)
Annual rate of change ADAS-Cog13	1.8 ± 4.4 [-5.6, 11.7] (75.1 %)	1.7 ± 4.5 [-5.7, 12.0] (96.7 %)	/	2.4 ± 3.7 [-4.3, 10.0]	/

(e) Early AD with high brain tau levels (ADAS-Cog13)

	ADNI
Number of forecasts	488
Number of subjects	170
Number of forecasts per subject	2.9 ± 2.0 [1, 8]
Trial duration (y)	4.4 ± 0.3 [4.0, 5.0]
Age at screening	72.0 ± 5.9 [58.1, 80.5]
Female	41.6%
**Education level**	
≤ 9 years	1.6%
Between 10 and 15 years	38.5%
≥ 16 years	59.8%
**APOE-ε4 copies**	
0	41.2%
1	44.7%
2	14.1%
**A/T/N/C profile at inclusion**	
A+T+N-C~	16.4%
A+T+N+C~	53.3%
A-T+ *	30.3%
**Score distribution at inclusion**	
CDR (global)	0.5
CDR-SB	1.5 ± 1.0 [0.0, 4.0]
MMSE	27.4 ± 2.2 [22.0, 30.0]
ADAS-Cog13	15.9 ± 6.8 [4.7, 28.6]
**ARC distribution**	
Annual rate of change CDR-SB	0.5 ± 0.7 [-0.3, 2.3] (98.4 %)
Annual rate of change MMSE	-0.8 ± 1.1 [-3.9, 0.5]
Annual rate of change ADAS-Cog13	2.0 ± 2.8 [-1.6, 9.7]

(f) MCI probably due to AD or mild AD (MMSE)

	Pooled	ADNI	AIBL	J-ADNI	MEMENTO	PHARMACOG
Number of forecasts	8,570	3,040	165	343	4,996	26
Number of subjects	2,193	711	102	174	1,192	14
Number of forecasts per subject	3.9 ± 2.7 [1, 10]	4.3 ± 2.9 [1, 11]	1.6 ± 0.5 [1, 2]	2.0 ± 0.2 [1, 2]	4.2 ± 2.7 [1, 9]	1.9 ± 1.0 [1, 3]
Trial duration (y)	2.9 ± 0.4 [2.3, 3.6]	3.0 ± 0.4 [2.3, 3.6]	3.0 ± 0.0 [3.0, 3.0]	2.8 ± 0.3 [2.5, 3.0]	2.9 ± 0.4 [2.3, 3.6]	2.6 ± 0.2 [2.5, 3.0]
Age at screening	72.6 ± 7.0 [58.3, 84.7]	73.6 ± 7.0 [59.6, 85.0]	74.2 ± 7.0 [61.0, 85.2]	73.5 ± 5.8 [62.3, 83.2]	71.9 ± 7.1 [57.8, 84.5]	72.3 ± 5.6 [64.4, 82.2]
Female	50.7 %	38.5 %	49.1 %	55.1 %	57.9 %	57.7 %
**Education level**						
≤ 9 years	14.1 %	1.5 %	23.0 %	12.2 %	21.5 %	53.8 %
Between 10 and 15 years	46.3 %	33.4 %	53.3 %	60.9 %	52.9 %	30.8 %
≥ 16 years	37.8 %	65.1 %	23.6 %	26.8 %	22.5 %	15.4 %
Missing	1.8 %				3.1 %	
**APOE-ε4 copies**						
0	57.9 %	52.0 %	45.5 %	45.8 %	63.0 %	30.8 %
1	32.1 %	36.5 %	38.2 %	44.9 %	28.1 %	65.4 %
2	7.6 %	11.4 %	16.4 %	8.7 %	4.9 %	3.8 %
Missing	2.4 %			0.6 %	4.0 %	
**A/T/N/C profile at inclusion**						
A*T*N*C~	57.6%	20.8%	84.8%	60.6%	79.1%	
A+T+N+C~	12.7%	23.4%		18.4%	6.2%	34.6%
A*T*N*C+	2.0%	2.3%	13.3%	2.6%	1.4%	
A+T+N+C+	1.3%	3.8%			0.0%	
A+[T- or N-]C[~ or +]	9.6%	21.0%	1.2%	7.6%	2.9%	53.8%
A-T+ *	4.7%	8.5%	0.6%	2.9%	2.7%	7.7%
A-T*N*C[~ or +]	11.9%	20.3%		7.9%	7.5%	3.8%
**Score distribution at inclusion**						
CDR (global)	0.5 ± 0.1 [0.5, 1.0]	0.5 ± 0.1 [0.5, 1.0]	0.6 ± 0.2 [0.5, 1.0]	0.5 ± 0.1 [0.5, 1.0]	0.5 ± 0.1 [0.5, 0.5]	0.5 ± 0.0 [0.5, 0.5]
CDR-SB	1.4 ± 1.2 [0.5, 5.0] (95.7 %)	1.9 ± 1.4 [0.5, 5.5]	2.0 ± 1.8 [0.5, 6.0] (99.4 %)	N.C.	1.1 ± 0.9 [0.5, 4.0]	/
MMSE	27.3 ± 2.5 [21.0, 30.0]	27.2 ± 2.7 [21.0, 30.0]	25.5 ± 3.7 [18.0, 30.0]	26.1 ± 2.2 [21.0, 30.0]	27.5 ± 2.2 [22.0, 30.0]	27.4 ± 1.8 [24.0, 30.0]
ADAS-Cog13	16.5 ± 8.2 [3.3, 34.0] (35.7 %)	16.4 ± 8.2 [3.3, 34.0] (99.8 %)	/	N.C.	/	19.6 ± 4.1 [11.3, 25.8]
**ARC distribution**						
Annual rate of change CDR-SB	0.4 ± 0.9 [-0.5, 2.9] (95.9 %)	0.5 ± 0.9 [-0.5, 3.0] (98.2 %)	1.0 ± 1.2 [-0.3, 4.0] (99.4 %)	1.1 ± 1.2 [-0.2, 4.3] (98.3 %)	0.2 ± 0.8 [-0.6, 2.6] (94.7 %)	/
Annual rate of change MMSE	-0.5 ± 1.3 [-4.0, 1.2]	-0.7 ± 1.4 [-4.4, 1.2]	-1.3 ± 1.9 [-6.0, 1.0]	-1.3 ± 1.5 [-5.5, 0.9]	-0.4 ± 1.1 [-3.5, 1.2]	-0.5 ± 0.8 [-1.6, 1.2]
Annual rate of change ADAS-Cog13	1.8 ± 3.1 [-2.3, 10.3] (39.0 %)	1.7 ± 3.1 [-2.4, 10.4] (97.9 %)	/	2.7 ± 2.9 [-1.7, 9.8]	/	0.6 ± 1.7 [-1.9, 3.3]

Supplementary Table 9 The MEMENTO study group

Name	Degree	Location	Role
Michèle Allard	MD, PhD	Memory Resource and Research Centre of Bordeaux, CHU de Bordeaux, Hôpital Xavier Arnozan, F-33000, Bordeaux, France	Co-investigator
Sandrine Andrieu	MD, PhD	Memory Resource and Research Centre of Toulouse, CHU de Toulouse, Hôpital La Grave-Casselardit, F-31000, Toulouse, France	Co-investigator
Pierre Anthony	MD, PhD	Memory Resource and Research Centre of Colmar, Hôpitaux Civils de Colmar, F-68000, Colmar, France	Co-investigator
Christine Astier	MD	Memory Resource and Research Centre of Strasbourg, Hôpitaux Universitaires de Strasbourg, F-67000, Strasbourg, France	Co-investigator
Alexandre Augier	MD, PhD	Memory Clinic, Hôpital Avicenne, AP-HP, Hôpitaux Universitaires Paris-Seine-Saint-Denis, F-93009, Bobigny, France	Co-investigator
Nicolas Auguste	MD	Memory Resource and Research Centre of Saint-Etienne, CHU de Saint-Etienne, Hôpital de la Charité, F-42000, Saint-Etienne, France	Co-investigator
Sophie Auriacombe	MD, PhD	Memory Resource and Research Centre of Bordeaux, CHU de Bordeaux, Hôpital Pellegrin, F-33000, Bordeaux, France	Co-investigator
John Avet	MD, PhD	Memory Resource and Research Centre of Saint-Etienne, CHU de Saint-Etienne, Hôpital Nord, F-42000, Saint-Etienne, France	Co-investigator
Olivier Bailon	MD, PhD	Memory Clinic, Hôpital Avicenne, AP-HP, Hôpitaux Universitaires Paris-Seine-Saint-Denis, F-93009, Bobigny, France	Co-investigator
Fabrice-Guy Barral	MD	Memory Resource and Research Centre of Saint-Etienne, CHU de Saint-Etienne, Hôpital Nord, F-42000, Saint-Etienne, France	Co-investigator
Jean Barré	MD	Memory Resource and Research Centre of Angers, CHU d'Angers, F-49000, Angers	Co-investigator
Annick Barthelaix	MD, PhD	Memory Resource and Research Centre of Angers, CHU d'Angers, F-49000, Angers	Co-investigator
Catherine Bayle	MD	Memory Resource and Research Centre of Paris Broca, AP-HP, Paris, France	Co-investigator
Olivier Beauchet		Memory Resource and Research Centre of Angers, CHU d'Angers, F-49000, Angers	Co-investigator
Catherine Belin	MD, PhD	Memory Clinic, Hôpital Avicenne, AP-HP, Hôpitaux Universitaires Paris-Seine-Saint-Denis, F-93009, Bobigny, France	Co-investigator
Samia Belkacem	MD	Institute of Memory and Alzheimer's Disease (IM2A), Centre for NeuroImaging Research (CENIR), Brain and Spine Institute (ICM), UMR S 1127, Department of Neurology, AP-HP, Pitié-Salpêtrière University Hospital, Sorbonne Universities, Pierre et Marie Curie University, Paris, France	Co-investigator
Douraied Ben Salem	MD, PhD	Memory Resource and Research Centre of Brest, CHRU de Brest, F-29000, Brest, France	Co-investigator
Karim Bennys	MD	Memory Resource and Research Centre of Montpellier, CHU de Montpellier, Hôpital Gui de Chauliac, F-34000, Montpellier, France	Co-investigator
Géraldine Bera	MD	Laboratoire d'Imagerie Biomédicale, Sorbonne Universités, UPMC Univ Paris 06, Inserm U1146, CNRS UMR 7371, France NeuroSpin, I2BM, Commissariat à l'Energie Atomique, Paris, France	Co-investigator
Eric Berger	MD	Memory Resource and Research Centre of Besançon, CHU de Besançon, Hôpital Jean Minjoz, Hôpital Saint-Jacques, F-25000, Besançon, France	Co-investigator
Marc G Berger	MD, PhD	Memory Resource and Research Centre of Clermont-Ferrand, CHU de Clermont-Ferrand, F-63000, Clermont-Ferrand, France	Co-investigator
Emilie Bergouin	MD	Memory Resource and Research Centre of Dijon, CHU Dijon Bourgogne, Hôpital du Bocage, Hôpital de Champmaillot, F-21000, Dijon, France	Co-investigator
François Bertin-Hugault	MD	Memory Resource and Research Centre of Lyon, Hospices Civils de Lyon, Hôpital des Charpennes, F-69000, Lyon, France	Co-investigator
Guillaume Bertrand	MD	Memory Clinic, Hôpital Avicenne, AP-HP, Hôpitaux Universitaires Paris-Seine-Saint-Denis, F-93009, Bobigny, France	Co-investigator
François-Xavier Bertrand	MD, PhD	Memory Resource and Research Centre of Nantes, CHU de Nantes, F-44000, Nantes, France	Co-investigator
Catherine Beze	MD	Memory Resource and Research Centre of Center Region, CHRU de Tours, Hôpital Bretonneau, F-37000, Tours, France	Co-investigator
Valérie Boilet		Coordinating Centre, Inserm CIC-1401 Clinical Epidemiology, CHU de Bordeaux, F-33000, Bordeaux, France	Co-investigator
Stéphanie Bombois	MD, PhD	Memory Resource and Research Centre of Lille, CHRU de Lille, Hôpital Roger Salengro, F-59000, Lille, France	Co-investigator
Alain Bonafé	MD, PhD	Memory Resource and Research Centre of Montpellier, CHU de Montpellier, Montpellier, France	Co-investigator
Yasmina Boudali	MD	Memory Resource and Research Centre of Paris Broca, AP-HP, Paris, France	Co-investigator
Hatem Bouhladour	MD, PhD	Memory Resource and Research Centre of Besançon, CHU de Besançon, Hôpital Jean Minjoz, Hôpital Saint-Jacques, F-25000, Besançon, France	Co-investigator
Clémence Boully	MD	Memory Resource and Research Centre of Paris Broca, AP-HP, Paris, France	Co-investigator

Isabelle Bourdel-Marchasson	MD, PhD	Memory Resource and Research Centre of Bordeaux, CHU de Bordeaux, Hôpital Xavier Arnozan, F-33000, Bordeaux, France	Co-investigator
Vincent Bouteloup	PharmD	Coordinating Centre, Inserm CIC-1401 Clinical Epidemiology, CHU de Bordeaux, F-33000, Bordeaux, France	Co-investigator
Claire Boutet	MD	Institute of Memory and Alzheimer's Disease (IM2A), Centre for NeuroImaging Research (CENIR), Brain and Spine Institute (ICM), UMR S 1127, Department of Neurology, AP-HP, Pitié-Salpêtrière University Hospital, Sorbonne Universities, Pierre et Marie Curie University, Paris, France	Co-investigator
Serge Bracard	MD, PhD	Memory Resource and Research Centre of Nancy, CHU de Nancy, F-54000, Nancy, France	Co-investigator
Antoine Brangier	MD	Memory Resource and Research Centre of Angers, CHU d'Angers, F-49000, Angers	Co-investigator
Pierre-Yves Brillet	MD, PhD	Memory Clinic, Hôpital Avicenne, AP-HP, Hôpitaux Universitaires Paris-Seine-Saint-Denis, F-93009, Bobigny, France	Co-investigator
Laure Caillard	MD	Memory Resource and Research Centre of Paris Broca, AP-HP, Paris, France	Co-investigator
Fabienne Calvas	MD	Memory Resource and Research Centre of Toulouse, CHU de Toulouse, Hôpital Purpan, F-31000, Toulouse, France	Co-investigator
Agnès Camus	MD	Memory Resource and Research Centre of Dijon, CHU Dijon Bourgogne, Hôpital du Bocage, Hôpital de Champmaillot, F-21000, Dijon, France	Co-investigator
Vincent Camus	MD, PhD	Memory Resource and Research Centre of Center Region, CHRU de Tours, Hôpital Bretonneau, F-37000, Tours, France	Co-investigator
Sandrine Canaple	MD	Memory Resource and Research of Amiens, CHU Amiens Picardie, F-80000, Amiens, France	Co-investigator
Antoine Carpentier	MD, PhD	Memory Clinic, Hôpital Avicenne, AP-HP, Hôpitaux Universitaires Paris-Seine-Saint-Denis, F-93009, Bobigny, France	Co-investigator
Pascaline Cassagnaud	MD	Memory Resource and Research Centre of Lille, CHRU de Lille, Hôpital Roger Salengro, F-59000, Lille, France	Co-investigator
Françoise Cattin	MD	Memory Resource and Research Centre of Besançon, CHU de Besançon, Hôpital Jean Minjoz, Hôpital Saint-Jacques, F-25000, Besançon, France	Co-investigator
Ludivine Chamard	MD	Memory Resource and Research Centre of Besançon, CHU de Besançon, Hôpital Jean Minjoz, Hôpital Saint-Jacques, F-25000, Besançon, France	Co-investigator
Stéphane Chanalet	MD	Memory Resource and Research Centre of Nice, CHU de Nice, Hôpital Pasteur, F-06100, Nice, France	Co-investigator
Mathieu Chastan	MD	Memory Resource and Research Centre of Rouen, CLCC Henri Becquerel, Rouen, France	Co-investigator
Sophie Chauvelier	MD	Memory Resource and Research Centre of Paris Broca, AP-HP, Paris, France	Co-investigator
Valérie Chauvire	MD	Memory Resource and Research Centre of Angers, CHU d'Angers, F-49000, Angers	Co-investigator
Samia Cheriet	MD, PhD	Memory Resource and Research Centre of Toulouse, CHU de Toulouse, Hôpital Purpan, F-31000, Toulouse, France	Co-investigator
Anthony Clotagatide	MD	Memory Resource and Research Centre of Saint-Etienne, CHU de Saint-Etienne, Hôpital Nord, F-42000, Saint-Etienne, France	Co-investigator
Emmanuel Cognat	MD, PhD	Memory Resource and Research Centre of Paris Nord, AP-HP, Paris, France	Co-investigator
Lora Cohen	PhD	Memory Resource and Research Centre of Grenoble, CHU de Grenoble Alpes, Grenoble, France	Co-investigator
Jean-Marc Constans	MD, PhD	Memory Resource and Research of Amiens, CHU Amiens Picardie, F-80000, Amiens, France	Co-investigator
Marie-Hélène Coste	MD, PhD	Memory Resource and Research Centre of Lyon, Hospices Civils de Lyon, Hôpital des Charpennes, F-69000, Lyon, France	Co-investigator
Jean-Philippe Cottier	MD, PhD	Memory Resource and Research Centre of Center Region, CHRU de Tours, Hôpital Bretonneau, F-37000, Tours, France	Co-investigator
François Cotton	MD, PhD	Memory Resource and Research Centre of Lyon, Hospices Civils de Lyon, Hôpital des Charpennes, F-69000, Lyon, France	Co-investigator
Isabelle Couret	MD	Memory Resource and Research Centre of Montpellier, CHU de Montpellier, Hôpital Gui de Chauliac, F-34000, Montpellier, France	Co-investigator
Olivier-François Couturier	MD, PhD	Memory Resource and Research Centre of Angers, CHU d'Angers, F-49000, Angers	Co-investigator
Pascale Cowppli-Bony	MD, PhD	Memory Resource and Research Centre of Bordeaux, CHU de Bordeaux, Hôpital Pellegrin, F-33000, Bordeaux, France	Co-investigator
Véronique Cressot	MD	Memory Resource and Research Centre of Bordeaux, CHU de Bordeaux, Hôpital Xavier Arnozan, F-33000, Bordeaux, France	Co-investigator
Benjamin Crétin	MD	Memory Resource and Research Centre of Strasbourg, Hôpitaux Universitaires de Strasbourg, F-67000, Strasbourg, France	Co-investigator
Keren Danaila	MD	Memory Resource and Research Centre of Lyon, Hospices Civils de Lyon, Hôpital des Charpennes, F-69000, Lyon, France	Co-investigator
Jacques Darcourt	MD, PhD	Memory Resource and Research Centre of Nice, CLCC Antoine Lacassagne, Nice, France	Co-investigator

Jean-François Dartigues	MD, PhD	Memory Resource and Research Centre of Bordeaux, CHU de Bordeaux, Hôpital Pellegrin, F-33000, Bordeaux, France	Co-investigator
Ana-Maria Dascalita	MD, PhD	Memory Resource and Research Centre of Saint-Etienne, CHU de Saint-Etienne, Hôpital de la Charité, F-42000, Saint-Etienne, France	Co-investigator
Renaud David	MD, PhD	Memory Resource and Research Centre of Nice, CHU de Nice, Institut Claude Pompidou, F-06100, Nice, France	Co-investigator
Xavier De Petigny	MD	Memory Resource and Research Centre of Strasbourg, Hôpitaux Universitaires de Strasbourg, F-67000, Strasbourg, France	Co-investigator
Delphine De Verbizier-Lonjon	MD	Memory Resource and Research Centre of Montpellier, CHU de Montpellier, Hôpital Gui de Chauliac, F-34000, Montpellier, France	Co-investigator
Marielle Decousus	MD, PhD	Memory Resource and Research Centre of Saint-Etienne, CHU de Saint-Etienne, Hôpital Nord, F-42000, Saint-Etienne, France	Co-investigator
Isabelle Defouilloy	MD, PhD	Memory Resource and Research of Amiens, CHU Amiens Picardie, F-80000, Amiens, France	Co-investigator
Christine Delmaire	MD, PhD	Memory Resource and Research Centre of Lille, CHRU de Lille, Hôpital Roger Salengro, F-59000, Lille, France	Co-investigator
Julien Delrieu	MD	Memory Resource and Research Centre of Toulouse, CHU de Toulouse, Hôpital La Grave-Casselardit, F-31000, Toulouse, France	Co-investigator
Catherine Demuyinck	MD	Memory Resource and Research Centre of Strasbourg, Hôpitaux Universitaires de Strasbourg, F-67000, Strasbourg, France	Co-investigator
Vincent Deramecourt	MD, PhD	Memory Resource and Research Centre of Lille, CHRU de Lille, Hôpital Roger Salengro, F-59000, Lille, France	Co-investigator
Hervé Deramond	MD, PhD	Memory Resource and Research of Amiens, CHU Amiens Picardie, F-80000, Amiens, France	Co-investigator
Thomas Desmidt	MD, PhD	Memory Resource and Research Centre of Center Region, CHRU de Tours, Hôpital Bretonneau, F-37000, Tours, France	Co-investigator
Marie-Dominique Desruet	PharmD, PhD	Memory Resource and Research Centre of Grenoble, CHU de Grenoble Alpes, Grenoble, France	Co-investigator
Julien Detour		Memory Resource and Research Centre of Strasbourg, Hôpitaux Universitaires de Strasbourg, F-67000, Strasbourg, France	Co-investigator
Agnès Devendeville	MD	Memory Resource and Research of Amiens, CHU Amiens Picardie, F-80000, Amiens, France	Co-investigator
Mira Didic	MD, PhD	Memory Resource and Research Centre of Marseille, CHU de Marseille, Hôpital La Timone, F-13000, Marseille, France	Co-investigator
Maritchu Doireau	MD	Memory Resource and Research Centre of Bordeaux, CHU de Bordeaux, Hôpital Pellegrin, F-33000, Bordeaux, France	Co-investigator
Antonio Dos Santos	MD	Institute of Memory and Alzheimer's Disease (IM2A), Brain and Spine Institute (ICM), UMR S 1127, Department of Neurology, AP-HP, Pitié-Salpêtrière University Hospital, Sorbonne Universities, Pierre et Marie Curie University, Paris, France	Co-investigator
Patrice Douillet	MD	Memory Resource and Research Centre of Montpellier, CHU de Montpellier, Hôpital Gui de Chauliac, F-34000, Montpellier, France	Co-investigator
Foucaud Du Boisgueheneuc	MD	Memory Resource and Research Centre of Poitiers, CHU de Poitiers, Hôpital de La Milétrie, F-86000, Poitiers, France	Co-investigator
Delphine Dubail	MD	Memory Resource and Research Centre of Paris Broca, AP-HP, Paris, France	Co-investigator
Laure Ducroq-Ducastaing	MD	Memory Resource and Research Centre of Brest, CHRU de Brest, F-29000, Brest, France	Co-investigator
Julien Dumurgier	MD, PhD	Memory Resource and Research Centre of Paris Nord, AP-HP, Paris, France	Co-investigator
Diane Dupuy	MD, PhD	Memory Resource and Research of Amiens, CHU Amiens Picardie, F-80000, Amiens, France	Co-investigator
Emmanuelle Duron	MD, PhD	Memory Resource and Research Centre of Paris Broca, AP-HP, Paris, France	Co-investigator
Inna Dygai-Cochet	MD, PhD	Memory Resource and Research Centre of Dijon, CLCC Georges François Leclerc, Dijon, France	Co-investigator
Véronique Eder	MD, PhD	Memory Clinic, Hôpital Avicenne, AP-HP, Hôpitaux Universitaires Paris-Seine-Saint-Denis, F-93009, Bobigny, France	Co-investigator
Stéphane Epelbaum	MD, PhD	Institute of Memory and Alzheimer's Disease (IM2A), Brain and Spine Institute (ICM), UMR S 1127, Department of Neurology, AP-HP, Pitié-Salpêtrière University Hospital, Sorbonne Universities, Pierre et Marie Curie University, Paris, France	Co-investigator
Frédérique Etcharry-Bouyx	MD, PhD	Memory Resource and Research Centre of Angers, CHU d'Angers, F-49000, Angers	Co-investigator
Daniel Fagret	MD, PhD	Memory Resource and Research Centre of Grenoble, CHU de Grenoble Alpes, Grenoble, France	Co-investigator
Catherine Faisant	MD	Memory Resource and Research Centre of Toulouse, CHU de Toulouse, Hôpital La Grave-Casselardit, F-31000, Toulouse, France	Co-investigator
Karim Farid	MD, PhD	Memory Resource and Research Centre of Paris Nord, AP-HP, Paris, France	Co-investigator
Denis Fédérico	MD	Memory Resource and Research Centre of Lyon, Hospices Civils de Lyon, Hôpital des Charpennes, F-69000, Lyon, France	Co-investigator

Olivier Felician	MD, PhD	Memory Resource and Research Centre of Marseille, CHU de Marseille, Hôpital La Timone, F-13000, Marseille, France	Co-investigator
Philippe Fernandez	MD, PhD	Memory Resource and Research Centre of Bordeaux, CHU de Bordeaux, Hôpital Pellegrin, F-33000, Bordeaux, France	Co-investigator
Pacôme Fosse	MD	Memory Resource and Research Centre of Angers, CHU d'Angers, F-49000, Angers	Co-investigator
Alexandra Foubert-Samier	MD, PhD	Memory Resource and Research Centre of Bordeaux, CHU de Bordeaux, Hôpital Pellegrin, F-33000, Bordeaux, France	Co-investigator
Isabelle Franck	MD	Memory Resource and Research Centre of Strasbourg, Hôpitaux Universitaires de Strasbourg, F-67000, Strasbourg, France	Co-investigator
Monique Galitzky	MD	Memory Resource and Research Centre of Toulouse, CHU de Toulouse, Hôpital Purpan, F-31000, Toulouse, France	Co-investigator
Céline Gallazzini-Crepin	MD	Memory Resource and Research Centre of Grenoble, CHU de Grenoble Alpes, Grenoble, France	Co-investigator
Radka Gantchev	MD	Memory Resource and Research Centre of Marseille, CHU de Marseille, Hôpital La Timone, F-13000, Marseille, France	Co-investigator
Laurence Garbarg-Chenon	MD	Memory Clinic, Hôpital Avicenne, AP-HP, Hôpitaux Universitaires Paris-Seine-Saint-Denis, F-93009, Bobigny, France	Co-investigator
Guillaume Gautier	MD, PhD	Memory Resource and Research Centre of Marseille, CHU de Marseille, Hôpital La Timone, F-13000, Marseille, France	Co-investigator
Emmanuel Gerardin	MD, PhD	Memory Resource and Research Centre of Rouen, Neuroradiology Department, Rouen University Hospital, F-76031, Rouen, France	Co-investigator
Claire Gervais	MD	Memory Resource and Research Centre of Nice, CHU de Nice, Institut Claude Pompidou, F-06100, Nice, France	Co-investigator
Jean-Claude Getenet	MD	Memory Resource and Research Centre of Saint-Etienne, CHU de Saint-Etienne, Hôpital Nord, F-42000, Saint-Etienne, France	Co-investigator
Nadine Girard	MD, PhD	Memory Resource and Research Centre of Marseille, CHU de Marseille, Hôpital La Timone, F-13000, Marseille, France	Co-investigator
Fabienne Giraud	MD	Memory Resource and Research Centre of Marseille, CHU de Marseille, Hôpital La Timone, F-13000, Marseille, France	Co-investigator
Chantal Girtanner	MD	Memory Resource and Research Centre of Saint-Etienne, CHU de Saint-Etienne, Hôpital de la Charité, F-42000, Saint-Etienne, France	Co-investigator
Valérie Gissot	MD	Memory Resource and Research Centre of Center Region, CHRU de Tours, Hôpital Bretonneau, F-37000, Tours, France	Co-investigator
Caroline Grangeon	PharmD	Memory Resource and Research Centre of Nice, CHU de Nice, Institut Claude Pompidou, F-06100, Nice, France	Co-investigator
Daniel Grucker	MD, PhD	Memory Resource and Research Centre of Strasbourg, Hôpitaux Universitaires de Strasbourg, F-67000, Strasbourg, France	Co-investigator
Eric Guedj	MD, PhD	Memory Resource and Research Centre of Marseille, CHU de Marseille, Hôpital La Timone, F-13000, Marseille, France	Co-investigator
Claude Gueriot	MD	Memory Resource and Research Centre of Marseille, CHU de Marseille, Hôpital La Timone, F-13000, Marseille, France	Co-investigator
Yves Guilhermet	MD	Memory Resource and Research Centre of Lyon, Hospices Civils de Lyon, Hôpital des Charpennes, F-69000, Lyon, France	Co-investigator
Rémy Guillevin	MD, PhD	Memory Resource and Research Centre of Poitiers, CHU de Poitiers, Hôpital de La Milétrie, F-86000, Poitiers, France	Co-investigator
Sophie Haffen	MD	Memory Resource and Research Centre of Besançon, CHU de Besançon, Hôpital Jean Minjoz, Hôpital Saint-Jacques, F-25000, Besançon, France	Co-investigator
Didier Hannequin	MD, PhD	Memory Resource and Research Centre of Rouen, Neurology Department, Rouen University Hospital, F-76031, Rouen, France	Co-investigator
Sandrine Harston	MD	Memory Resource and Research Centre of Bordeaux, CHU de Bordeaux, Hôpital Xavier Arnozan, F-33000, Bordeaux, France	Co-investigator
Anne Hitzel	MD, PhD	Memory Resource and Research Centre of Toulouse, CHU de Toulouse, Hôpital Purpan, F-31000, Toulouse, France	Co-investigator
Caroline Hommet	MD, PhD	Memory Resource and Research Centre of Center Region, CHRU de Tours, Hôpital Bretonneau, F-37000, Tours, France	Co-investigator
Claude Hossein-Foucher	MD, PhD	Memory Resource and Research Centre of Lille, CHRU de Lille, Hôpital Roger Salengro, F-59000, Lille, France	Co-investigator
Fabrice Hubele	MD	Memory Resource and Research Centre of Strasbourg, Hôpitaux Universitaires de Strasbourg, F-67000, Strasbourg, France	Co-investigator
Agnès Jacquin-Piques	MD, PhD	Memory Resource and Research Centre of Dijon, CHU Dijon Bourgogne, Hôpital du Bocage, Hôpital de Champmaillot, F-21000, Dijon, France	Co-investigator
Betty Jean	MD	Memory Resource and Research Centre of Clermont-Ferrand, CHU de Clermont-Ferrand, F-63000, Clermont-Ferrand, France	Co-investigator
Joanne Jenn	MD, PhD	Memory Resource and Research Centre of Bordeaux, CHU de Bordeaux, Hôpital Xavier Arnozan, F-33000, Bordeaux, France	Co-investigator
Laure Joly	MD, PhD	Memory Resource and Research Centre of Nancy, CHU de Nancy, F-54000, Nancy, France	Co-investigator
Thérèse Jonveaux	MD	Memory Resource and Research Centre of Nancy, CHU de Nancy, F-54000, Nancy, France	Co-investigator

Adrien Julian	MD, PhD	Memory Resource and Research Centre of Poitiers, CHU de Poitiers, Hôpital de La Milétrie, F-86000, Poitiers, France	Co-investigator
Aurélie Kas	MD, PhD	Laboratoire d'Imagerie Biomédicale, Sorbonne Universités, UPMC Univ Paris 06, Inserm U1146, CNRS UMR 7371, France NeuroSpin, I2BM, Commissariat à l'Energie Atomique, Paris, France	Co-investigator
Anna Kearney-Schwartz	MD	Memory Resource and Research Centre of Nancy, CHU de Nancy, F-54000, Nancy, France	Co-investigator
Alice Keles	MD	Memory Resource and Research Centre of Nancy, CHU de Nancy, F-54000, Nancy, France	Co-investigator
Antony Kelly	MD	Memory Resource and Research Centre of Clermont-Ferrand, Centre de Lutte contre le Cancer, F-63000, Clermont-Ferrand, France	Co-investigator
Nathalie Keromnes	MD	Memory Resource and Research Centre of Brest, CHRU de Brest, F-29000, Brest, France	Co-investigator
Lejla Koric	MD	Memory Resource and Research Centre of Marseille, CHU de Marseille, Hôpital La Timone, F-13000, Marseille, France	Co-investigator
Alexandre Krainik	MD, PhD	Memory Resource and Research Centre of Grenoble, CHU de Grenoble Alpes, Grenoble, France	Co-investigator
Stéphane Kremer	MD	Memory Resource and Research Centre of Strasbourg, Hôpitaux Universitaires de Strasbourg, F-67000, Strasbourg, France	Co-investigator
Florian Labourée	MD	Memory Resource and Research Centre of Paris Broca, AP-HP, Paris, France	Co-investigator
Franck Lacoeuille	MD, PhD	Memory Resource and Research Centre of Angers, CHU d'Angers, F-49000, Angers	Co-investigator
Francoise Lala	MD	Memory Resource and Research Centre of Toulouse, CHU de Toulouse, Hôpital La Grave-Casselardit, F-31000, Toulouse, France	Co-investigator
Chantal Lamy	MD	Memory Resource and Research of Amiens, CHU Amiens Picardie, F-80000, Amiens, France	Co-investigator
Jean-Louis Laplanche	PharmD, PhD	Memory Resource and Research Centre of Paris Nord, AP-HP, Paris, France	Co-investigator
Cyrille Launay	MD, PhD	Memory Resource and Research Centre of Angers, CHU d'Angers, F-49000, Angers	Co-investigator
Stéphane Lehericy	MD, PhD	Institute of Memory and Alzheimer's Disease (IM2A), Centre for NeuroImaging Research (CENIR), Brain and Spine Institute (ICM), UMR S 1127, Department of Neurology, AP-HP, Pitié-Salpêtrière University Hospital, Sorbonne Universities, Pierre et Marie Curie University, Paris, France	Co-investigator
Sylvain Lehmann	MD, PhD	Memory Resource and Research Centre of Montpellier, CHU de Montpellier, Hôpital Gui de Chauliac, F-34000, Montpellier, France	Co-investigator
Hermine Lenoir	MD, PhD	Memory Resource and Research Centre of Paris Broca, AP-HP, Paris, France	Co-investigator
Marcel Levy	MD, PhD	Institute of Memory and Alzheimer's Disease (IM2A), Brain and Spine Institute (ICM), UMR S 1127, Department of Neurology, AP-HP, Pitié-Salpêtrière University Hospital, Sorbonne Universities, Pierre et Marie Curie University, Paris, France	Co-investigator
Stéphanie Libercier	MD, PhD	Memory Resource and Research Centre of Colmar, Hôpitaux Civils de Colmar, F-68000, Colmar, France	Co-investigator
Marie-Anne Mackowiak-Cordoliani	MD	Memory Resource and Research Centre of Lille, CHRU de Lille, Hôpital Roger Salengro, F-59000, Lille, France	Co-investigator
Eloi Magnin	MD	Memory Resource and Research Centre of Besançon, CHU de Besançon, Hôpital Jean Minjoz, Hôpital Saint-Jacques, F-25000, Besançon, France	Co-investigator
Zaza Makaroff	MD	Memory Resource and Research Centre of Lyon, Hospices Civils de Lyon, Hôpital des Charpennes, F-69000, Lyon, France	Co-investigator
Athina Marantidou	MD	Memory Clinic, Hôpital Avicenne, AP-HP, Hôpitaux Universitaires Paris-Seine-Saint-Denis, F-93009, Bobigny, France	Co-investigator
Isabelle Marcet	MD	Memory Resource and Research Centre of Bordeaux, CHU de Bordeaux, Hôpital Pellegrin, F-33000, Bordeaux, France	Co-investigator
Cécilia Marelli	MD, PhD	Memory Resource and Research Centre of Montpellier, CHU de Montpellier, Hôpital Gui de Chauliac, F-34000, Montpellier, France	Co-investigator
Sophie Marilier	MD	Memory Resource and Research Centre of Dijon, CHU Dijon Bourgogne, Hôpital du Bocage, Hôpital de Champmaillot, F-21000, Dijon, France	Co-investigator
Idalie Martin	MD	Memory Resource and Research Centre of Lyon, Hospices Civils de Lyon, Hôpital des Charpennes, F-69000, Lyon, France	Co-investigator
Olivier Martinaud	MD, PhD	Memory Resource and Research Centre of Rouen, Neurology Department, Rouen University Hospital, F-76031, Rouen, France	Co-investigator
Catherine Martin-Hunyadi	MD	Memory Resource and Research Centre of Strasbourg, Hôpitaux Universitaires de Strasbourg, F-67000, Strasbourg, France	Co-investigator
Aïcha Medjoul	MD	Memory Clinic, Hôpital Avicenne, AP-HP, Hôpitaux Universitaires Paris-Seine-Saint-Denis, F-93009, Bobigny, France	Co-investigator
Isabelle Merlet	MD	Memory Resource and Research Centre of Poitiers, CHU de Poitiers, Hôpital de La Milétrie, F-86000, Poitiers, France	Co-investigator
Danielle Mestas	MD	Memory Resource and Research Centre of Clermont-Ferrand, CHU de Clermont-Ferrand, F-63000, Clermont-Ferrand, France	Co-investigator

Marc-Etienne Meyer	MD, PhD	Memory Resource and Research of Amiens, CHU Amiens Picardie, F-80000, Amiens, France	Co-investigator
Jean-Marc Michel	MD	Memory Resource and Research Centre of Colmar, Hôpitaux Civils de Colmar, F-68000, Colmar, France	Co-investigator
Agnès Michon	MD	Institute of Memory and Alzheimer's Disease (IM2A), Brain and Spine Institute (ICM), UMR S 1127, Department of Neurology, AP-HP, Pitié-Salpêtrière University Hospital, Sorbonne Universities, Pierre et Marie Curie University, Paris, France	Co-investigator
Isabelle Migeon-Duballet	MD	Memory Resource and Research Centre of Poitiers, CHU de Poitiers, Hôpital de La Milétrie, F-86000, Poitiers, France	Co-investigator
Karl Mondon	MD, PhD	Memory Resource and Research Centre of Center Region, CHRU de Tours, Hôpital Bretonneau, F-37000, Tours, France	Co-investigator
Clément Morgat	PharmD, PhD	Memory Resource and Research Centre of Bordeaux, CHU de Bordeaux, Hôpital Pellegrin, F-33000, Bordeaux, France	Co-investigator
Véronique Moullart	MD	Memory Resource and Research of Amiens, CHU Amiens Picardie, F-80000, Amiens, France	Co-investigator
Christian Moussard	MD	Memory Resource and Research Centre of Besançon, CHU de Besançon, Hôpital Jean Minjoz, Hôpital Saint-Jacques, F-25000, Besançon, France	Co-investigator
Aurélie Mouton	MD, PhD	Memory Resource and Research Centre of Nice, CHU de Nice, Institut Claude Pompidou, F-06100, Nice, France	Co-investigator
Izzie Jacques Namer	MD, PhD	Memory Resource and Research Centre of Strasbourg, Hôpitaux Universitaires de Strasbourg, F-67000, Strasbourg, France	Co-investigator
Georges Niewiadomski	MD, PhD	Memory Resource and Research Centre of Nice, CHU de Nice, Institut Claude Pompidou, F-06100, Nice, France	Co-investigator
Guillaume Nivaggioni	MD	Memory Resource and Research Centre of Nice, CHU de Nice, Institut Claude Pompidou, F-06100, Nice, France	Co-investigator
Marie Noblet	MD, PhD	Memory Resource and Research Centre of Strasbourg, Hôpitaux Universitaires de Strasbourg, F-67000, Strasbourg, France	Co-investigator
Michel Nonent	MD, PhD	Memory Resource and Research Centre of Brest, CHRU de Brest, F-29000, Brest, France	Co-investigator
Fati Nourhashemi	MD, PhD	Memory Resource and Research Centre of Toulouse, CHU de Toulouse, Hôpital La Grave-Casselardit, F-31000, Toulouse, France	Co-investigator
Hélène Oesterle	MD	Memory Resource and Research Centre of Colmar, Hôpitaux Civils de Colmar, F-68000, Colmar, France	Co-investigator
Galdric Orvoen	MD	Memory Resource and Research Centre of Paris Broca, AP-HP, Paris, France	Co-investigator
Pierre Jean Ousset	MD, PhD	Memory Resource and Research Centre of Toulouse, CHU de Toulouse, Hôpital La Grave-Casselardit, F-31000, Toulouse, France	Co-investigator
Amandine Pallardy	MD	Memory Resource and Research Centre of Nantes, CHU de Nantes, F-44000, Nantes, France	Co-investigator
Claire Paquet	MD, PhD	Memory Resource and Research Centre of Paris Nord, AP-HP, Paris, France	Co-investigator
Pierre-Yves Pare	MD, PhD	Memory Resource and Research Centre of Angers, CHU d'Angers, F-49000, Angers	Co-investigator
Anne Pasco	MD, PhD	Memory Resource and Research Centre of Angers, CHU d'Angers, F-49000, Angers	Co-investigator
Pierre Payoux	MD, PhD	Memory Resource and Research Centre of Toulouse, CHU de Toulouse, Hôpital Purpan, F-31000, Toulouse, France	Co-investigator
Cécile Pays	MD, PhD	Memory Resource and Research Centre of Montpellier, CHU de Montpellier, Hôpital Gui de Chauliac, F-34000, Montpellier, France	Co-investigator
Isabelle Pellegrin	MD, PhD	Biological Research Centre, CHU de Bordeaux, F-33000, Bordeaux, France	Co-investigator
Rémy Perdrisot	MD, PhD	Memory Resource and Research Centre of Poitiers, CHU de Poitiers, Hôpital de La Milétrie, F-86000, Poitiers, France	Co-investigator
Bertille Perin	MD, PhD	Memory Resource and Research of Amiens, CHU Amiens Picardie, F-80000, Amiens, France	Co-investigator
Christine Perret-Guillaume	MD, PhD	Memory Resource and Research Centre of Nancy, CHU de Nancy, F-54000, Nancy, France	Co-investigator
Grégory Petyt	MD	Memory Resource and Research Centre of Lille, CHRU de Lille, Hôpital Roger Salengro, F-59000, Lille, France	Co-investigator
Nathalie Philippi	MD, PhD	Memory Resource and Research Centre of Strasbourg, Hôpitaux Universitaires de Strasbourg, F-67000, Strasbourg, France	Co-investigator
Geneviève Pinganaud	MD	Memory Resource and Research Centre of Bordeaux, CHU de Bordeaux, Hôpital Xavier Arnozan, F-33000, Bordeaux, France	Co-investigator
Matthieu Plichart	MD	Memory Resource and Research Centre of Paris Broca, AP-HP, Paris, France	Co-investigator
Gabriel Pop	MD, PhD	Memory Clinic, Hôpital Avicenne, AP-HP, Hôpitaux Universitaires Paris-Seine-Saint-Denis, F-93009, Bobigny, France	Co-investigator
Michèle Puel	MD	Memory Resource and Research Centre of Toulouse, CHU de Toulouse, Hôpital Purpan, F-31000, Toulouse, France	Co-investigator
Mathieu Queneau	MD, PhD	Memory Resource and Research Centre of Paris Nord, Centre Cardiologique du Nord, Paris, France	Co-investigator
Solène Querellou	MD	Memory Resource and Research Centre of Brest, CHRU de Brest, F-29000, Brest, France	Co-investigator

Muriel Quillard-Muraine	MD, PhD	Memory Resource and Research Centre of Rouen, Neurology Department, Rouen University Hospital, F-76031, Rouen, France	Co-investigator
Valérie Quipourt	MD, PhD	Memory Resource and Research Centre of Dijon, CHU Dijon Bourgogne, Hôpital du Bocage, Hôpital de Champmaillot, F-21000, Dijon, France	Co-investigator
Chloé Rachez	MD, PhD	Memory Resource and Research Centre of Clermont-Ferrand, CHU de Clermont-Ferrand, F-63000, Clermont-Ferrand, France	Co-investigator
Micheline Razzouk-Cadet	MD	Memory Resource and Research Centre of Nice, CHU de Nice, Institut Claude Pompidou, F-06100, Nice, France	Co-investigator
Anne-Sophie Rigaud	MD, PhD	Memory Resource and Research Centre of Paris Broca, AP-HP, Paris, France	Co-investigator
Hélène Robin-Ismer	MD	Memory Resource and Research Centre of Strasbourg, Hôpitaux Universitaires de Strasbourg, F-67000, Strasbourg, France	Co-investigator
Mathieu Rodallec	MD, PhD	Memory Resource and Research Centre of Paris Nord, Centre Cardiologique du Nord, Paris, France	Co-investigator
Yves Rolland	MD, PhD	Memory Resource and Research Centre of Toulouse, CHU de Toulouse, Hôpital La Grave-Casselardit, F-31000, Toulouse, France	Co-investigator
Adeline Rollin-Sillaire	MD, PhD	Memory Resource and Research Centre of Lille, CHRU de Lille, Hôpital Roger Salengro, F-59000, Lille, France	Co-investigator
Olivier Rouaud	MD	Memory Resource and Research Centre of Dijon, CHU Dijon Bourgogne, Hôpital du Bocage, Hôpital de Champmaillot, F-21000, Dijon, France	Co-investigator
Caroline Roubaud	MD, PhD	Memory Resource and Research Centre of Lyon, Hospices Civils de Lyon, Hôpital des Charpennes, F-69000, Lyon, France	Co-investigator
Isabelle Rouch	MD, PhD	Memory Resource and Research Centre of Lyon, Hospices Civils de Lyon, Hôpital des Charpennes, F-69000, Lyon, France	Co-investigator
Julie Roux	MD, PhD	Memory Resource and Research Centre of Grenoble, CHU de Grenoble Alpes, Grenoble, France	Co-investigator
Guillaume Sacco	MD, PhD	Memory Resource and Research Centre of Nice, CHU de Nice, Institut Claude Pompidou, F-06100, Nice, France	Co-investigator
Pierre-Yves Salaun	MD	Memory Resource and Research Centre of Brest, CHRU de Brest, F-29000, Brest, France	Co-investigator
François Salmon	MD, PhD	Memory Resource and Research Centre of Poitiers, CHU de Poitiers, Hôpital de La Milétrie, F-86000, Poitiers, France	Co-investigator
Alicia Sanchez	MD	Memory Resource and Research Centre of Saint-Etienne, CHU de Saint-Etienne, Hôpital Nord, F-42000, Saint-Etienne, France	Co-investigator
Maria-Joao Santiago-Ribeiro	MD, PhD	Memory Resource and Research Centre of Center Region, CHRU de Tours, Hôpital Bretonneau, F-37000, Tours, France	Co-investigator
Alain Sarciron	MD	Memory Resource and Research Centre of Lyon, Hospices Civils de Lyon, Hôpital des Charpennes, F-69000, Lyon, France	Co-investigator
Nathalie Sastre-Hengan	MD	Memory Resource and Research Centre of Toulouse, CHU de Toulouse, Hôpital La Grave-Casselardit, F-31000, Toulouse, France	Co-investigator
Mathilde Sauvée	MD, PhD	Memory Resource and Research Centre of Grenoble, CHU de Grenoble Alpes, Grenoble, France	Co-investigator
Christian Scheiber	MD, PhD	Memory Resource and Research Centre of Lyon, Hospices Civils de Lyon, Hôpital des Charpennes, F-69000, Lyon, France	Co-investigator
Anne-Marie Schneider	MD, PhD	Memory Resource and Research Centre of Strasbourg, Hôpitaux Universitaires de Strasbourg, F-67000, Strasbourg, France	Co-investigator
Franck Semah	MD, PhD	Memory Resource and Research Centre of Lille, CHRU de Lille, Hôpital Roger Salengro, F-59000, Lille, France	Co-investigator
Amélie Serra	MD	Memory Resource and Research Centre of Grenoble, CHU de Grenoble Alpes, Grenoble, France	Co-investigator
Marie-Laure Seux	MD	Memory Resource and Research Centre of Paris Broca, AP-HP, Paris, France	Co-investigator
Hélène Sordet-Guépet	MD	Memory Resource and Research Centre of Dijon, CHU Dijon Bourgogne, Hôpital du Bocage, Hôpital de Champmaillot, F-21000, Dijon, France	Co-investigator
Maria Eugenia Soto	MD	Memory Resource and Research Centre of Toulouse, CHU de Toulouse, Hôpital La Grave-Casselardit, F-31000, Toulouse, France	Co-investigator
Mathieu Tafani	MD	Memory Resource and Research Centre of Toulouse, CHU de Toulouse, Hôpital Purpan, F-31000, Toulouse, France	Co-investigator
Jean-Yves Tanguy	MD, PhD	Memory Resource and Research Centre of Angers, CHU d'Angers, F-49000, Angers	Co-investigator
Michael Taroux	MD, PhD	Memory Resource and Research Centre of Dijon, CHU Dijon Bourgogne, Hôpital du Bocage, Hôpital de Champmaillot, F-21000, Dijon, France	Co-investigator
Marc Teichmann	MD, PhD	Institute of Memory and Alzheimer's Disease (IM2A), Brain and Spine Institute (ICM), UMR S 1127, Department of Neurology, AP-HP, Pitié-Salpêtrière University Hospital, Sorbonne Universities, Pierre et Marie Curie University, Paris, France	Co-investigator
Catherine Terrat	MD, PhD	Memory Resource and Research Centre of Saint-Etienne, CHU de Saint-Etienne, Hôpital de la Charité, F-42000, Saint-Etienne, France	Co-investigator
Jamila Thabet	MD	Memory Clinic, Hôpital Avicenne, AP-HP, Hôpitaux Universitaires Paris-Seine-Saint-Denis, F-93009, Bobigny, France	Co-investigator

Claire Thalamas	MD	Memory Resource and Research Centre of Toulouse, CHU de Toulouse, Hôpital Purpan, F-31000, Toulouse, France	Co-investigator
Catherine Thomas-Anterion	MD, PhD	Memory Resource and Research Centre of Saint-Etienne, CHU de Saint-Etienne, Hôpital Nord, F-42000, Saint-Etienne, France	Co-investigator
Anne-Cécile Troussière	MD	Memory Resource and Research Centre of Lille, CHRU de Lille, Hôpital Roger Salengro, F-59000, Lille, France	Co-investigator
Renata Ursu	MD	Memory Clinic, Hôpital Avicenne, AP-HP, Hôpitaux Universitaires Paris-Seine-Saint-Denis, F-93009, Bobigny, France	Co-investigator
Pierre Vera	MD, PhD	Memory Resource and Research Centre of Rouen, CLCC Henri Becquerel, Rouen, France	Co-investigator
Martine Vercelletto	MD	Memory Resource and Research Centre of Nantes, CHU de Nantes, F-44000, Nantes, France	Co-investigator
Olivier Vercruysse	MD	Memory Resource and Research Centre of Lille, CHRU de Lille, Hôpital Roger Salengro, F-59000, Lille, France	Co-investigator
Antoine Verger	MD, PhD	Memory Resource and Research Centre of Nancy, CHU de Nancy, F-54000, Nancy, France	Co-investigator
Philippe Viau	MD	Memory Resource and Research Centre of Nice, CHU de Nice, Institut Claude Pompidou, F-06100, Nice, France	Co-investigator
Marie-Neige Videau	MD	Memory Resource and Research Centre of Bordeaux, CHU de Bordeaux, Hôpital Xavier Arnozan, F-33000, Bordeaux, France	Co-investigator
Thierry Voisin	MD	Memory Resource and Research Centre of Toulouse, CHU de Toulouse, Hôpital La Grave-Casselardit, F-31000, Toulouse, France	Co-investigator
Nathalie Wagemann	MD, PhD	Memory Resource and Research Centre of Nantes, CHU de Nantes, F-44000, Nantes, France	Co-investigator
Aziza Waissi-Sediq	MD	Memory Resource and Research Centre of Lyon, Hospices Civils de Lyon, Hôpital des Charpennes, F-69000, Lyon, France	Co-investigator
Jing Xie	MD, PhD	Memory Resource and Research Centre of Lyon, Hospices Civils de Lyon, Hôpital des Charpennes, F-69000, Lyon, France	Co-investigator
Nathanaëlle Yeni	MD	Laboratoire d'Imagerie Biomédicale, Sorbonne Universités, UPMC Univ Paris 06, Inserm U1146, CNRS UMR 7371, France NeuroSpin, I2BM, Commissariat à l'Energie Atomique, Paris, France	Co-investigator
Michel Zanca	MD, PhD	Memory Resource and Research Centre of Montpellier, CHU de Montpellier, Hôpital Gui de Chauliac, F-34000, Montpellier, France	Co-investigator
Jean Zinszner	MD, PhD	Memory Clinic, Hôpital Avicenne, AP-HP, Hôpitaux Universitaires Paris-Seine-Saint-Denis, F-93009, Bobigny, France	Co-investigator
