# Supplementary material for: Forecasting individual progression trajectories in Alzheimer’s disease
Source: Nat Commun. 2023 Feb 10;14:761. doi: 10.1038/s41467-022-35712-5 (PMC9918533; doi:10.1038/s41467-022-35712-5)
Supplement: Supplementary file 3 — Reporting Summary [file 41467_2022_35712_MOESM3_ESM.pdf]

nature portfolio

Corresponding author(s): Stanley Durrleman

Last updated by author(s): Nov 22, 2022

Reporting Summary

Nature Portfolio wishes to improve the reproducibility of the work that we publish. This form provides structure for consistency and transparency in reporting. For further information on Nature Portfolio policies, see our Editorial Policies and the Editorial Policy Checklist.

Statistics

For all statistical analyses, confirm that the following items are present in the figure legend, table legend, main text, or Methods section.

n/a	Confirmed	
☐	☒	The exact sample size (n) for each experimental group/condition, given as a discrete number and unit of measurement
☐	☒	A statement on whether measurements were taken from distinct samples or whether the same sample was measured repeatedly
☐	☒	The statistical test(s) used AND whether they are one- or two-sided<br>*Only common tests should be described solely by name; describe more complex techniques in the Methods section.*
☐	☒	A description of all covariates tested
☐	☒	A description of any assumptions or corrections, such as tests of normality and adjustment for multiple comparisons
☐	☒	A full description of the statistical parameters including central tendency (e.g. means) or other basic estimates (e.g. regression coefficient) AND variation (e.g. standard deviation) or associated estimates of uncertainty (e.g. confidence intervals)
☐	☒	For null hypothesis testing, the test statistic (e.g. F, t, r) with confidence intervals, effect sizes, degrees of freedom and P value noted<br>*Give P values as exact values whenever suitable.*
☐	☒	For Bayesian analysis, information on the choice of priors and Markov chain Monte Carlo settings
☒	☐	For hierarchical and complex designs, identification of the appropriate level for tests and full reporting of outcomes
☐	☒	Estimates of effect sizes (e.g. Cohen's d, Pearson's r), indicating how they were calculated

Our web collection on statistics for biologists contains articles on many of the points above.

Software and code

Policy information about availability of computer code

Data collection	We used already collected data. No software was used for data collection in this study.
Data analysis	Data analysis was done using the open-source software leapsy, accessible on the following GitLab repository: https://gitlab.com/icm-institute/aramislab/leaspy.<br><br>The version of the software that was used is available at the following Zenodo repository https://zenodo.org/record/7331109.

For manuscripts utilizing custom algorithms or software that are central to the research but not yet described in published literature, software must be made available to editors and reviewers. We strongly encourage code deposition in a community repository (e.g. GitHub). See the Nature Portfolio guidelines for submitting code & software for further information.

Data

Policy information about availability of data

All manuscripts must include a data availability statement. This statement should provide the following information, where applicable:

- Accession codes, unique identifiers, or web links for publicly available datasets
- A description of any restrictions on data availability
- For clinical datasets or third party data, please ensure that the statement adheres to our policy

The ADNI and AIBL data used in this study are available in the database of the laboratory of neuroimaging at the university of Southern California under accession code at http://adni.loni.usc.edu.

The J-ADNI data used in this study are available in the NBDC Human Database under accession code at http://humandbs.biosciencedbc.jp/en/.

The PharmaCog data used in this study are available in the NeuGRID2 platform under access code at https://www.neugrid2.eu/ (https://doi.org/10.17616/R31NJN1E)

The MEMENTO data used in this study are available in Dementia Platform UK under accession code at https://portal.dementiasplatform.uk/CohortDirectory/Item?fingerPrintID=MEMENTO

Raw data and patient-level data that were generated in this study are protected and are not available due to data privacy laws and data use agreements. These data can be re-generated using the open-source software Leaspy by anyone with an authorized access to the above third-party data. The data used to compute the statistics in this study are available at: https://zenodo.org/record/7331109. Source data are provided with this paper.

Field-specific reporting

Please select the one below that is the best fit for your research. If you are not sure, read the appropriate sections before making your selection.

[x] Life sciences [ ] Behavioural & social sciences [ ] Ecological, evolutionary & environmental sciences

For a reference copy of the document with all sections, see nature.com/documents/nr-reporting-summary-flat.pdf

Life sciences study design

All studies must disclose on these points even when the disclosure is negative.

Sample size	No statistical method was used to predetermine sample size. We considered all available data from all the cohorts. The resulting sample size is much larger than comparable observational studies.
Data exclusions	We only excluded participants with less than 1 year of follow-up. These participants could not be used to train the model, nor to assess forecast errors.
Replication	We performed a repeated 5-fold cross-validation procedure on the ADNI data set, and used 4 other independent data sets as external validation sets. Forecasts errors in the test sets were never statistically greater than the errors in the training set, thus showing generalizability across studies.
Randomization	The experiments were not randomized since only observational data were used. Simulations of clinical trials included a random unblinded allocation into treated and control arms.
Blinding	The investigators were not blinded to allocation during experiments and outcome assessment since only observational data were used. Simulations of clinical trials included a random unblinded allocation into treated and control arms with assessment of biases in sex, center, level of education and APOE genotype.

Reporting for specific materials, systems and methods

We require information from authors about some types of materials, experimental systems and methods used in many studies. Here, indicate whether each material, system or method listed is relevant to your study. If you are not sure if a list item applies to your research, read the appropriate section before selecting a response.

Materials & experimental systems

n/a	Involved in the study	
x		Antibodies
x		Eukaryotic cell lines
x		Palaeontology and archaeology
x		Animals and other organisms
	x	Human research participants
x		Clinical data
x		Dual use research of concern

Methods

n/a	Involved in the study	
x		ChIP-seq
x		Flow cytometry
	x	MRI-based neuroimaging

Human research participants

Policy information about studies involving human research participants

Population characteristics	N=4,687 participants with an average of 6 visits per participants and 4 years of follow-up. Average age at baseline: 71,7 years. 54,2% female participants, 39% of APOE epsilon 4 carriers. At baseline, N=1,985 participants were cognitively unimpaired, N=2,347 experienced mild cognitive impairment and N=355 had a diagnostic of dementia.
Recruitment	Participants were recruited in memory clinics in North America, Europe, Australia and Japan. Recruitments relied on outreach efforts including online and social media campaigns, advertisement in magazines and radios, communications to healthcare provider and consumer advocacy organizations, use of registries, distribution of booklets and supporting communication materials to recruiting centers.<br><br>In general, minorities are under represented in clinical studies although ADNI 3 made specific efforts to include at least 12% of minority enrollment. Therefore, results might not generalize to under-represented minorities within each area (USA, Australia, Europe, Japan)
Ethics oversight	The study protocols were approved by the ethical committees of the university of southern California (ADNI), Austin Health, St Vincent's Health, Hollywook Private Hospital and Edith Cowan University (AIBL), IRCCS Istituto Centro San Giovanni di Dio Fatebenefratelli (PharmaCog), Comité de protection des personnes sud-ouest et outre-mer III (MEMENTO), the National Bioscience Database Center Human Database (J-ADNI).<br><br>Informed consent forms were obtained from research participants. The research has been performed in accordance with the Declaration of Helsinki and relevant guidelines and regulations. Participants were not compensated for the current study.

Note that full information on the approval of the study protocol must also be provided in the manuscript.

Magnetic resonance imaging

Experimental design

Design type	na
Design specifications	na
Behavioral performance measures	na

Acquisition

Imaging type(s)	structural and PET
Field strength	3T (ADNI 2, Go, 3, AIBL, PharmaCog, Memento) and 1.5T (AIBL (24% of the scans), Memento (14% of the scans), J-ADNI)
Sequence & imaging parameters	three-dimensional T1-weighted magnetization-prepared rapid gradient-echo imaging (MPRAGE) sequences
Area of acquisition	whole brain

Diffusion MRI ☐ Used ☒ Not used

Preprocessing

Preprocessing software	Freesurfer 5.3 and 6.0
Normalization	Internal Freesurfer procedure (see https://surfer.nmr.mgh.harvard.edu/ftp/articles/fischl02-labeling.pdf)
Normalization template	Internal Freesurfer probabilistic atlas (see https://surfer.nmr.mgh.harvard.edu/ftp/articles/fischl02-labeling.pdf)
Noise and artifact removal	Gradwarp, B1-correction, N3 bias field correction and geometric scaling for adjusting scanner- and session-specific calibration errors
Volume censoring	no volume censoring

Statistical modeling & inference

Model type and settings	Only subcortical volumes extracted from the MRI were used in the study. They were adjusted by estimated total intracranial volume.
Effect(s) tested	na

Specify type of analysis: ☐ Whole brain ☒ ROI-based ☐ Both

Anatomical location(s)	Automatic volume calculation of lateral ventricles, hippocampus and total intra-cranial volume from Freesurfer.
Statistic type for inference (See Eklund et al. 2016)	na
Correction	na

Models & analysis

n/a	Involved in the study
☒	☐ Functional and/or effective connectivity
☒	☐ Graph analysis
☐	☒ Multivariate modeling or predictive analysis

Multivariate modeling and predictive analysis

Variables included in the multivariate analysis: MMSE, ADASCog13, CDR-SB, sum of left and right hippocampus volume normalised by total intracranial volume, total volume of the lateral ventricles normalised by total intracranial volume, CSF beta-amyloid, total and phosphorylated tau levels, whole-brain standard uptake value ratio from [18F]AV-45 and [18F]AV-1451 PET scans.

No dimensional reduction was done.

Model used is a non-linear mixed-effect model.

Training metrics: goodness-of-fit.

Evaluation metrics: forecast errors on test participants with latest visits hidden during model personalisation. Area under the receiver operating characteristics curves (AUC), sensitivity, specificity, balanced accuracy, precision, negative predictive value for the automatic selection of participants.
